# Efficacy and safety of first-line immunotherapy and targeted therapy in advanced HCC: a network meta-analysis with subgroup analysis based on HBV and HCV infection

**DOI:** 10.3389/fimmu.2026.1706446

**Published:** 2026-01-29

**Authors:** Qinfei Li, Hong Li, Haowei Ma, Wei Chen

**Affiliations:** 1 Wisconsin School of Business, University of Wisconsin–Madison, Madison, WI, United States; 2 School of Pharmacy, Duquesne University, Pittsburgh, PA, United States; 3 Department of Mechanical and Aerospace Engineering, Case Western Reserve University, Cleveland, OH, United States; 4 Department of Pharmacy, Emergency General Hospital, Beijing, China

**Keywords:** bayesian network meta-analysis, first-line therapy, HBV, HCV, hepatocellular carcinoma, immune checkpoint inhibitors, targeted therapy

## Abstract

**Introduction:**

We conducted an etiology-stratified network meta-analysis of first-line systemic therapies for advanced HCC to compare newer regimens beyond sorafenib-based RCT evidence (HBV, HCV, or non-viral).

**Methods:**

Following PRISMA-NMA, we searched PubMed, Embase, Cochrane Library, and Web of Science to 01 June 2025 for first-line RCTs in advanced/unresectable HCC. Primary endpoints were overall survival (OS) and progression-free survival (PFS); secondary endpoints were objective response rate (ORR) and grade ≥3 adverse events (AEs≥3). A Bayesian fixed-effects NMA (gemtc v4.4 with rjags) reported hazard ratios (HRs) or risk ratios (RRs) with 95% credible intervals, calculated SUCRA values for ranking, and assessed network coherence using deviance information criterion differences between consistency and inconsistency models. Protocol registered in PROSPERO (CRD420251074687).

**Results:**

Twenty-four RCTs (n=13,572) evaluating 26 first-line regimens formed a connected evidence network. In the overall population, regimens with significant OS advantage over sorafenib included sintilimab plus bevacizumab biosimilar (HR = 0.57, 95% CrI 0.43–0.75), camrelizumab plus rivoceranib (HR = 0.62, 0.48–0.79), and atezolizumab plus bevacizumab (HR = 0.66, 0.51–0.84). For PFS, top-ranked combinations were camrelizumab plus rivoceranib (HR = 0.52, 0.41–0.66), anlotinib plus penpulimab (HR = 0.53, 0.41–0.68), lenvatinib plus pembrolizumab (HR = 0.55, 0.44–0.68), and sintilimab plus bevacizumab biosimilar (HR = 0.56, 0.45–0.69). ORR was highest with lenvatinib plus pembrolizumab (RR = 8.00, 4.98–12.86). Regarding safety, tislelizumab (RR = 0.42, 0.33–0.52) and nivolumab (RR = 0.45, 0.36–0.56) were associated with the lowest incidence of AEs≥3. Etiology-stratified analyses indicated that, in HBV-related HCC, sintilimab plus bevacizumab biosimilar and atezolizumab plus bevacizumab led OS rankings, with PFS favoring cabozantinib plus atezolizumab and atezolizumab plus bevacizumab. In HCV-related HCC, only atezolizumab plus bevacizumab conferred a significant OS benefit (HR = 0.43, 0.25–0.73), while PFS superiority was observed only for cabozantinib plus atezolizumab (HR = 0.73, 0.54–0.99). In non-viral HCC, the STRIDE regimen (single priming dose tremelimumab plus durvalumab) was the only regimen to significantly improve OS (HR = 0.75, 0.59–0.96).

**Conclusions:**

For first-line therapy in advanced HCC, ICI-based combinations with anti-VEGF/anti-angiogenic agents generally outperform sorafenib, with discernible etiology-specific optima: HBV-related HCC favors sintilimab plus bevacizumab biosimilar or atezolizumab plus bevacizumab; HCV-related HCC favors atezolizumab plus bevacizumab; and in non-viral disease, STRIDE demonstrates a unique OS advantage. This etiology-stratified evidence framework may guide individualized first-line decision-making, pending confirmation in head-to-head trials.

**Systematic review registration:**

https://www.crd.york.ac.uk/prospero/display_record.php?ID=CRD420251074687, CRD420251074687.

## 1 Introduction

Hepatocellular carcinoma (HCC) is the predominant histologic subtype of primary liver cancer, accounting for over 80% of cases and imposing a substantial global health burden (1). Its incidence shows pronounced geographic heterogeneity, with hyperendemic foci in East Asia and sub-Saharan Africa largely reflecting the high prevalence of chronic hepatitis B (HBV) and hepatitis C (HCV) infection in these regions (1). Accordingly, the fraction of HCC attributable to viral hepatitis is markedly higher in these settings, underscoring the central etiologic role of HBV/HCV in hepatocarcinogenesis (1). Beyond oncogenesis, viral etiology shapes the disease course and modulates responsiveness to systemic agents (2). Viral-associated and non-viral HCC differ in molecular features, baseline hepatic reserve, and, critically, the tumor immune microenvironment (TIME) (3). For example, HBV-related HCC commonly exhibits dense intrahepatic inflammatory infiltrates with marked T-cell exhaustion, whereas HCV-related HCC appears to rely on distinct immune-evasion programs (3). Consequently, etiology is a key determinant of the efficacy and safety of immunotherapies and targeted agents and should be explicitly considered in trial design and in the formulation of personalized treatment strategies.

Systemic therapy is the cornerstone of care for patients with advanced or unresectable HCC. For more than a decade, sorafenib, the first approved multi-targeted tyrosine kinase inhibitor (TKI), served as the benchmark first-line option. The seminal SHARP trial (2008) established it as the standard of care by demonstrating a significant improvement in overall survival (OS) versus placebo (10.7 vs 7.9 months) (4). Nevertheless, its overall benefit is modest: the objective response rate (ORR) is low (2-3%), and treatment-related adverse events, most commonly hand-foot skin reaction, hypertension, and diarrhea, are frequent; grade ≥3 toxicities are sufficiently common to prompt dose reductions or discontinuation. These tolerability issues can degrade quality of life and limit treatment persistence, thereby constraining effectiveness outside tightly controlled trial settings (4).

Recent advances in tumor immunology and molecular targeting have reshaped the therapeutic landscape of advanced HCC. Two drug classes have driven this shift: immune checkpoint inhibitors (ICIs) and newer multi-targeted TKIs. By relieving inhibitory signaling through PD-1/PD-L1 and CTLA-4, ICIs restore T-cell-mediated antitumor immunity and have produced durable clinical activity across multiple solid tumors (2). In parallel, next-generation TKIs have shown substantial therapeutic potential by potently suppressing angiogenic and proliferative signaling cascades pertinent to HCC biology (5). A wave of phase III randomized trials has reshaped the first-line treatment landscape for advanced HCC. Several regimens, typically pairing an ICI with an anti-angiogenic agent or a multi-targeted TKI, have shown superior efficacy to sorafenib and have broadened therapeutic options. In IMbrave150, atezolizumab plus bevacizumab significantly improved OS and PFS versus sorafenib, marking a paradigm shift in standard care (6). HIMALAYA subsequently validated dual-checkpoint blockade with the STRIDE regimen (tremelimumab plus durvalumab) (7), whereas COSMIC-312 evaluated cabozantinib plus atezolizumab and demonstrated less consistent benefits relative to sorafenib (8). On the strength of these data, several combinations have received regulatory approval for first-line use, and combination therapy has become the prevailing approach.

This rapid progress, however, has created a new clinical dilemma. With most pivotal trials using sorafenib as the comparator, direct head-to-head evidence among the newer regimens is scarce. This leaves clinicians without clear guidance on how to choose the best first-line therapy for their patients. Moreover, the role of viral etiology remains a critical but unresolved question. While many trials reported HBV- and HCV-stratified analyses, the findings have been inconsistent or underpowered, leaving a clear need for a comprehensive synthesis to guide treatment decisions. To resolve this uncertainty and provide clarity for clinicians, we conducted a comprehensive network meta-analysis (NMA) of all first-line phase III randomized controlled trials. Our primary goal was to establish a clear hierarchy of efficacy and safety across all contemporary regimens. A key secondary objective was to determine how treatment effects differ by viral etiology through stratified analyses for HBV and HCV. By integrating all available direct and indirect evidence, this NMA aims to build an evidence-based framework that supports more personalized, etiology-driven first-line treatment decisions.

## 2 Materials and methods

This NM) was designed and reported in accordance with the PRISMA extension for network meta-analyses; the completed checklist is provided in **Supplementary Table 1**. Given the paucity of head-to-head RCTs comparing contemporary immunotherapy-based regimens, we prespecified a Bayesian framework to synthesize direct and indirect evidence and to generate probabilistic treatment rankings for efficacy and safety. To ensure transparency and methodological rigor, the protocol was prospectively registered with PROSPERO (CRD420251074687). We conducted a systematic, comprehensive search of four electronic databases: PubMed, Embase, the Cochrane Library, and Web of Science from inception through June 1, 2025.

To ensure a comprehensive retrieval of all relevant literature, the search strategy for each database combined controlled vocabulary (e.g., MeSH, Emtree) with a broad range of free-text keywords, with no language restrictions applied. The search query was constructed using terms across three key domains: terms to identify the population (e.g., “hepatocellular carcinoma,” “HCC,” “liver cancer”); a comprehensive list of interventions, including individual drug names (e.g., “atezolizumab,” “lenvatinib”) and their mechanisms (e.g., “immune checkpoint inhibitors,” “PD-1”); and a validated, highly sensitive filter for study design to capture only randomized controlled trials. The full, database-specific search strategies are detailed in **Supplementary Table 2**.

### 2.2 Selection criteria

#### 2.2.1 Inclusion

1. Randomized controlled trials (RCTs) enrolling patients with histologically or cytologically confirmed advanced or unresectable HCC.
2. Studies evaluating first-line systemic therapy, including immune checkpoint inhibitors, targeted agents, or their combinations, with or without a prespecified locoregional modality (e.g., HAIC, TACE, SBRT) as part of the randomized first-line strategy.
3. Comparator arms consisting of sorafenib, lenvatinib, or any other accepted first-line systemic regimen.
4. Trials reporting at least one of the following outcomes:

OS: time from randomization to death from any cause; Progression-free survival (PFS): time from randomization to documented disease progression or death; ORR: proportion of patients achieving complete or partial response; Grade ≥3 adverse events (AEs): as defined by the Common Terminology Criteria for Adverse Events (CTCAE).

#### 2.2.2 Exclusion

1. Non-randomized designs, including observational studies, retrospective analyses, real-world evidence reports, single-arm trials, case reports, or narrative/systematic reviews.
2. Reports lacking extractable data for OS, PFS, ORR, or grade ≥3 AEs.
3. Duplicate publications, interim analyses, or secondary reports based on the same study population without additional usable data; only the most complete and most recent publication was retained.

Titles and abstracts were screened prior to full-text evaluation. All eligible RCTs underwent independent triple review by three investigators to ensure that the dataset reflected the latest publication for each study population.

### 2.3 Data extraction and quality assessment

**Figure 1:**
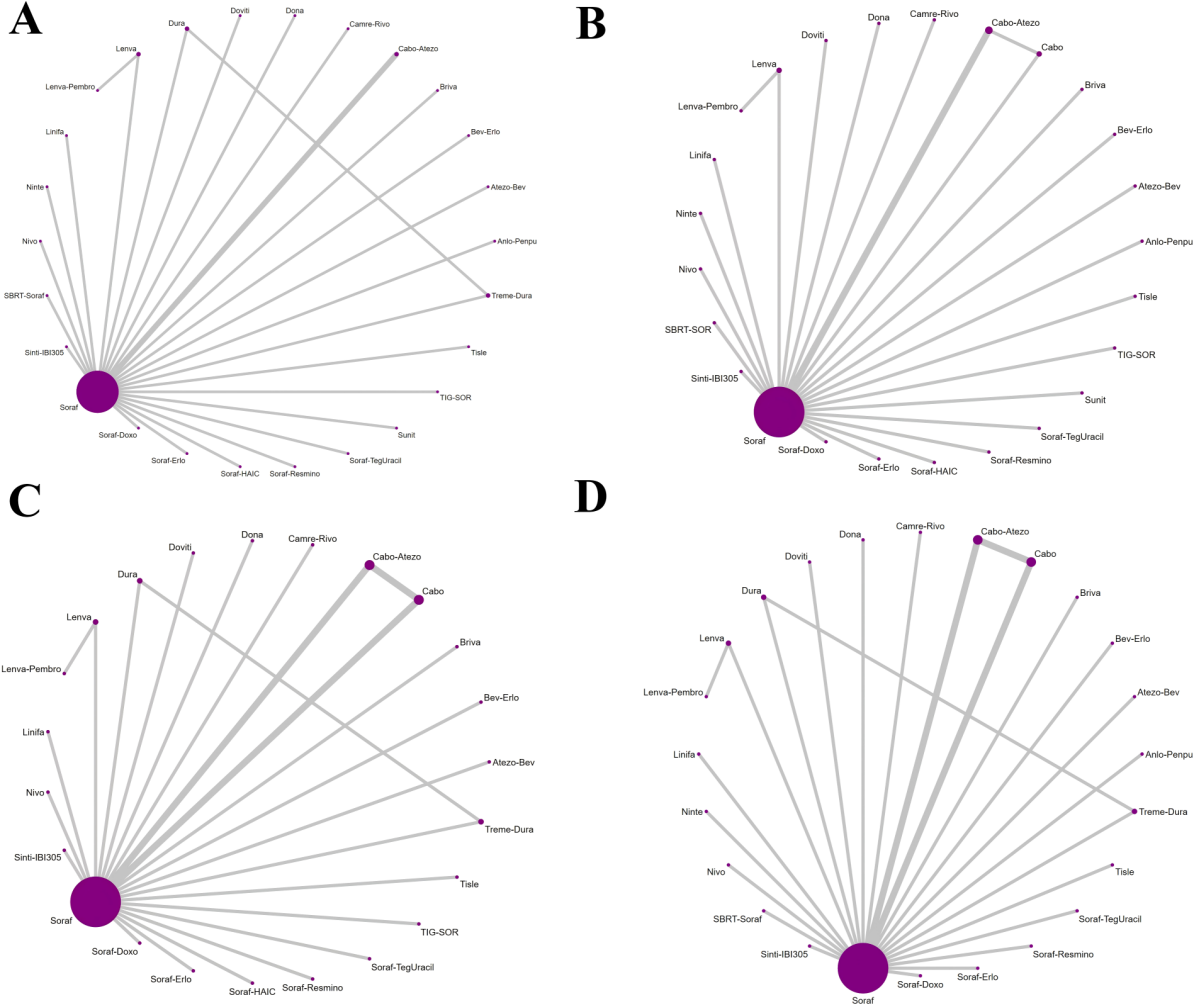
Network geometry of first-line immunotherapy and targeted therapy regimens for Overall Survival (OS), PFS, ORR, and Grade ≥3 AEs in the overall population of patients with advanced HCC. The specific networks are for **(A)** OS, **(B)** PFS, **(C)** ORR, and **(D)** AEs ≥3.

Three investigators independently extracted data from the included randomized controlled trials using a piloted, standardized form; disagreements were resolved by discussion and consensus, with adjudication by a fourth reviewer when required. Extracted items included trial-level characteristics (name/acronym, registry identifier [NCT], publication year, phase, randomization ratio, first-line treatment setting, disease type [HCC] with histologic/cytologic confirmation), patient characteristics (per-arm sample size and viral etiology [HBV/HCV], when reported), and intervention details (experimental and control regimens and planned doses for immunotherapy, targeted agents, or their combinations). For time-to-event endpoints, OS and PFS, we recorded hazard ratios (HRs) with 95% confidence intervals (CIs); for ORR and grade ≥ 3 adverse events (AEs), we extracted the number of events and the total number of patients per arm. Where available, HBV- and HCV-stratified data were also captured. The network geometry, depicting direct and indirect comparisons across all interventions, is shown in **Figure 1**. For the assessment of ORR, criteria varied slightly across older and newer studies. To ensure consistency across the network, data based on RECIST v1.1 were prioritized for extraction in all cases where multiple criteria were reported (e.g., REFLECT trial). For studies that only reported World Health Organization (WHO) criteria or mRECIST, those definitions were accepted but noted as a potential source of heterogeneity. A detailed summary of the response assessment criteria and review methods (e.g., Independent Review Committee vs. Investigator Assessment) for each included study is provided in **Supplementary Table S5**.

Methodological quality was appraised using the revised Cochrane Risk of Bias tool (RoB 2.0), which evaluates five domains: bias arising from the randomization process; deviations from intended interventions; missing outcome data; measurement of outcomes; and selection of the reported result. Each trial received an overall judgment of low risk, some concerns, or high risk.

### 2.4 Statistical analysis

The primary endpoints were OS and PFS. Secondary endpoints included ORR and the incidence of grade ≥3 adverse events (AEs). For efficacy outcomes, OS and PFS were summarized as hazard ratios (HRs) with 95% credible intervals (95% CIs); ORR and grade ≥3 AEs were expressed as risk ratios (RRs) with 95% CIs. Bayesian network meta-analysis. We conducted a Bayesian network meta-analysis in R using the gemtc (version 4.4) and rjags packages. Fixed-effect models were fitted for each outcome. We prespecified a fixed-effect Bayesian NMA because the evidence network was predominantly anchored by a common comparator and several interventions were informed by a single randomized trial, making the between-study variance difficult to estimate reliably and potentially overly driven by heterogeneity priors under a random-effects specification. In addition, the included studies were largely phase II/III first-line RCTs in advanced/unresectable HCC with broadly comparable eligibility criteria and endpoint definitions, which reduces the expectation of substantial between-trial heterogeneity for relative treatment effects. To assess the robustness of this assumption, we additionally examined random-effects models as a sensitivity analysis. For posterior estimation, we ran four parallel Markov chain Monte Carlo (MCMC) chains, discarded the first 20,000 iterations as burn-in, and then drew 50,000 additional sampling iterations to obtain posterior summaries. To compare treatments, we calculated the surface under the cumulative ranking curve (SUCRA) for both primary and secondary endpoints; SUCRA values closer to 1 indicate a more favorable efficacy or safety profile. Ranking probabilities for each treatment at every possible rank were derived from the posterior distribution using rank. Probability() and visualized as heatmaps with pheatmap. Global consistency of the network was assessed by comparing deviance information criterion (DIC) values between the consistency and inconsistency models; a DIC difference > 5 was interpreted as evidence of appreciable inconsistency.

## 3 Results

### 3.1 Systematic review and characteristics of the included studies

**Figure 2:**
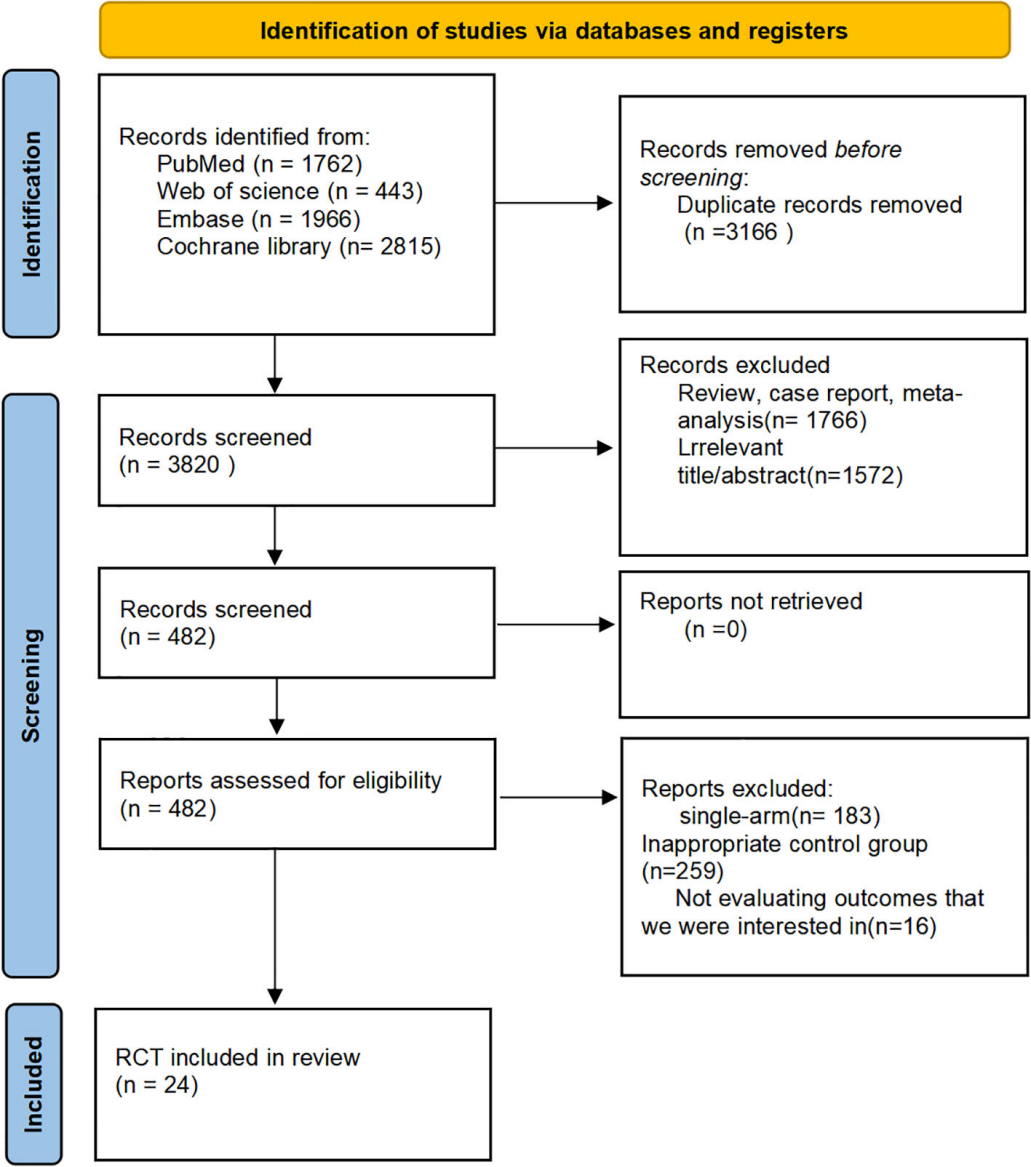
Flow diagram of the literature search and screening process in accordance with the PRISMA guidelines.

The literature search identified 6,986 records across four databases: PubMed (n=1,762), Cochrane Library (n=2,815), Embase (n=1,966), and Web of Science (n=443). After de-duplication, titles and abstracts were screened, and 482 articles underwent full-text assessment. Ultimately, 24 RCTs met the eligibility criteria and were included in the analysis (6–29). All evaluated first-line systemic regimens for advanced HCC and together formed a single connected evidence network suitable for network meta-analysis. The complete study-selection process is shown in the PRISMA flow diagram (**Figure 2**).

The included trials enrolled 13,572 participants. The evaluated regimens (network nodes) comprised: Durvalumab plus Tremelimumab (Treme-Dura), Durvalumab (Dura), Camrelizumab plus Rivoceranib (Camre-Rivo), Tislelizumab (Tisle), Lenvatinib plus Pembrolizumab (Lenva-Pembro), Cabozantinib plus Atezolizumab (Cabo-Atezo), Cabozantinib (Cabo), Anlotinib plus Penpulimab (Anlo-Penpu), Sintilimab plus Bevacizumab (Sinti-IBI305), Atezolizumab plus Bevacizumab (Atezo-Bev), Nivolumab (Nivo), Doxorubicin plus Sorafenib (Doxo-Soraf), Bevacizumab plus Erlotinib (Bev-Erlo), Resminostat plus Sorafenib (Soraf-Resmino), Nintedanib (Ninte), Sorafenib plus HAIC (Soraf-HAIC), Lenvatinib (Lenva), Sorafenib plus tegafur-uracil (UFT) (Soraf-TegUracil), Dovitinib (Doviti), Sorafenib plus Erlotinib (Soraf-Erlo), Linifanib (Linifa), Tigatuzumab plus Sorafenib (TIG-SOR), Brivanib (Briva), Sunitinib (Sunit), Stereotactic Body Radiation Therapy followed by Sorafenib (SBRT-Soraf), and Donafenib (Dona). Detailed trial characteristics are summarized **Tables 1**, **2** and **Supplementary Tables S5**, **S6**.

**Table 1:** Study-level baseline characteristics of trials included in the network meta-analysis.

Study	Source	Registered ID	Sample size	Stage (phase, design)	(y)	(Randomization)	(Median age/y)
HIMALAYA	Ann Oncol	NCT03298451	393/389/389	IV (open-label, phase III)	2024	1:1:1	66/63/64
CARES-310	JAMA Oncol	NCT03764293	272/271	IV (double-blind, phase III)	2023	1:1	58/56
RATIONALE-301	Lancet Oncol	NCT03412773	342/332	IV (open-label, phase III)	2023	1:1	62/60
LEAP-002	Lancet Oncol	NCT03713593	395/399	IV (double-blind, phase III)	2024	1:1	64/64
COSMIC-312	Lancet Oncol	NCT03755791	432/217/188	IV (double-blind, phase III)	2024	2:1:1	64/64/64
ALTN-AK105-III-02	Ann Oncol	NCT04344158	433/216	IV (open-label, phase III)	2024	2:1	NR
ORIENT-32	Lancet Oncol	NCT03794440	380/191	IV (double-blind, phase III)	2021	2:1:1	53/54
IMbrave 150	J Hepatol	NCT03434379	336/165	IV (open-label, phase III)	2022	2:1	NR
CheckMate 459	Lancet Oncol	NCT02576509	371/372	III (open-label, phase III)	2022	1:1	NR
CALGB 80802	JAMA Oncol	NCT01015833	180/176	III (unblinded, phase III)	2019	1:1	62/62
Thomas & Garrett-Mayer 2018	Oncology	NCT00881751	47/43	II (open-label, randomized phase II)	2018	1:1	61/61
Tak & Ryoo 2018	Invest New Drugs	NCT02400788	86/84	I/II (open-label; randomized phase II)	2018	1:1	65/62
Palmer & Ma 2018	Br J Cancer	NCT01004003	62/31	I/II (open-label; randomized phase II)	2018	2:1	66/64
SILIUS	Lancet Gastroenterol Hepatol	NCT01214343	103/102	III (open-label, phase III)	2018	1:1	68/69
REFLECT	Lancet	NCT01761266	478/476	III (open-label, non-inferiority phase III)	2018	1:1	61/61
ESLC01	J Hepatocellular Carcinoma	NCT01539018	36/38	II (open-label, randomized phase II)	2018	1:1	59/59
Cheng & Thongprasert 2016	Hepatology	NR	82/83	II (open-label, randomized phase II)	2016	1:1	56/56
SEARCH	J Clin Oncol	NCT00901901	362/358	III (double-blind, placebo-controlled phase III)	2014	1:1	61/60
Cainap & Qin 2014	J Clin Oncol	NCT01009593	514/521	III (open-label, randomized phase III; superiority/noninferiority)	2014	1:1	59/60
Cheng & Kang 2015	J Hepatol	NCT01033240	53/54/55	II (open-label, randomized phase II)	2015	1:1:1	63/63/66
BRISK-FL	J Clin Oncol	NCT00858871	578/577	III (double-blind, noninferiority phase III)	2013	1:1	60/61
SUN 1170	J Clin Oncol	NCT00699374	530/544	III (open-label, phase III; superiority/noninferiority hybrid)	2013	1:1	59/59
NRG/RTOG 1112	Int J Radiat Oncol Biol Phys	NCT01730937	92/85	III (open-label, phase III)	2022	1:1	66/66
Donafenib vs Sorafenib 2021	J Clin Oncol	NR	328/331	II–III (open-label, randomized, parallel-controlled)	2021	1:1	53/53

**Table 2:** Included RCTs and intervention regimens in the network meta-analysis.

Study	Intervention arm(s)	Control arm
HIMALAYA	300 mg of tremelimumab (one dose) plus 1500 mg of durvalumab every 4 weeks (STRIDE), 1500 mg of durvalumab mono therapy every 4 weeks	400 mg of sorafenib twice daily
CARES-310	camrelizumab 200 mg intravenously every 2 weeks plus rivoceranib 250 mg orally once daily	400 mg of sorafenib twice daily
RATIONALE-301	tislelizumab, 200 mg intravenously every 3 weeks	400 mg of sorafenib twice daily
LEAP-002	lenvatinib (body weight <60 kg, 8 mg/day; body weight ≥60 kg, 12 mg/day) plus pembrolizumab (200 mg every 3 weeks)	lenvatinib (body weight <60 kg, 8 mg/day; body weight ≥60 kg, 12 mg/day)
COSMIC-312	arm1:cabozantinib 40 mg once daily plus intravenous atezolizumab 1200 mg every 3 weeks arm2:cabozantinib 60 mg once daily	400 mg of sorafenib twice daily
ALTN-AK105-III-02	anlotinib (10 mg, po, qd, d1-14) plus penpulimab (200 mg, iv, q3w)	400 mg of sorafenib twice daily
ORIENT-32	200 mg of sintilimab intravenously over 60 min, followed by 15 mg/kg bodyweight of IBI305 bevacizumab biosimilar intravenously over 90 min (the second infusion over 60 min, and afterwards over 30 min if no infusion reaction occurred), every 3 weeks.	400 mg of sorafenib twice daily
IMbrave 150	1200 mg of atezolizumab plus 15 mg/kg of bevacizumab intravenously every 3 weeks	400 mg of sorafenib twice daily
CheckMate 459	nivolumab (240 mg intravenously every 2 weeks)	400 mg of sorafenib twice daily
CALGB 80802	Doxorubicin 60 mg/m² q21d + Sorafenib 400 mg BID	400 mg of sorafenib twice daily
Thomas & Garrett-Mayer 2018	Bevacizumab 10 mg/kg IV q14d + Erlotinib 150 mg QD	400 mg of sorafenib twice daily
Tak & Ryoo 2018	Resminostat 400 mg d1–5 q14d + Sorafenib 400 mg BID	400 mg of sorafenib twice daily
Palmer & Ma 2018	Nintedanib 200 mg BID	400 mg of sorafenib twice daily
SILIUS	Sorafenib 400 mg BID + HAIC (Cisplatin 20 mg/m² d1,8 + 5-FU 330 mg/m² d1–5 and 8–12 q28d)	400 mg of sorafenib twice daily
REFLECT	Lenvatinib 12 mg (≥60 kg) or 8 mg (<60 kg) QD	400 mg of sorafenib twice daily
ESLC01	Sorafenib 400 mg BID + UFT 125 mg/m² BID d1–28 q35d	400 mg of sorafenib twice daily
Cheng & Thongprasert 2016	Dovitinib 500 mg/day (5 days on, 2 off)	400 mg of sorafenib twice daily
SEARCH	Sorafenib 400 mg BID + Erlotinib 150 mg QD	400 mg of sorafenib twice daily
Cainap & Qin 2014	Linifanib 17.5 mg QD	400 mg of sorafenib twice daily
Cheng & Kang 2015	Tigatuzumab 6 mg/kg LD then 2 or 6 mg/kg weekly + Sorafenib 400 mg BID	400 mg of sorafenib twice daily
BRISK-FL	Brivanib 800 mg QD	400 mg of sorafenib twice daily
SUN 1170	Sunitinib 37.5 mg QD (continuous dosing)	400 mg of sorafenib twice daily
NRG/RTOG 1112	SBRT (27.5–50 Gy/5 fx; dose individualized to mean liver dose/OAR limits),Sorafenib 200 mg PO BID, escalate to 400 mg PO BID at Day 28 if appropriate.	400 mg of sorafenib twice daily
Donafenib vs Sorafenib 2021	Donafenib 0.2 g BID	400 mg of sorafenib twice daily

**Figure 3:**
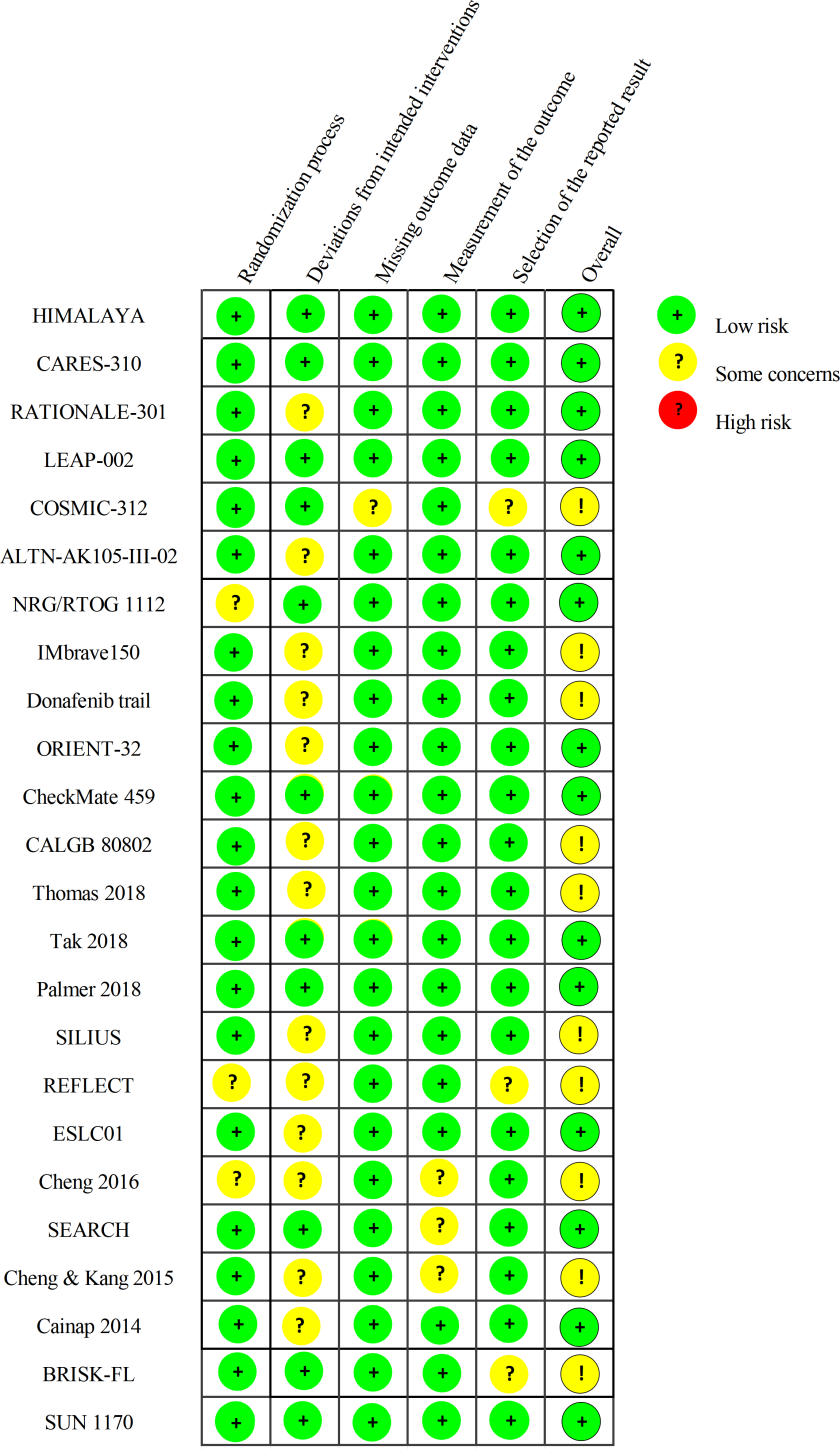
Summary of the RoB 2.0 assessment.

Risk of bias was assessed using the revised Cochrane tool (RoB 2.0). Of the 24 trials, 14 were judged low risk and 10 some concerns (**Figure 3**). Most “some concerns” judgments arose in the deviations from intended interventions domain owing to open-label designs. For example, in RATIONALE-301 (15), although OS was the primary endpoint, knowledge of assignment could plausibly have influenced investigator behavior (e.g., adverse-event management or treatment discontinuation). Similar considerations applied to ALTN-AK105-III-02 (10) and NCT00881751, where lack of blinding may have affected intervention delivery, particularly for more subjective outcomes. CheckMate 459 (11) was also open label, and early crossover to second-line therapy in some patients introduced potential postrandomization confounding for OS. In the nonblinded CALGB 80802 trial (29), awareness of treatment groups may have influenced toxicity management and dose-modification decisions. Additional concerns were identified for COSMIC-312 (8). In the missing outcome data domain, incompletely explained missingness, especially for subgroup and secondary endpoints, raised the possibility of differential attrition. In the selection of the reported result domain, several subgroup and secondary-endpoint analyses were not clearly prespecified in the trial registry, suggesting a risk of selective reporting.

### 3.2 Network meta-analyses

#### 3.2.1 Comparisons of OS, PFS and ORR

The primary outcomes were overall survival (OS) and progression-free survival (PFS); secondary outcomes were objective response rate (ORR) and adverse events of grade ≥3.

**Figure 4:**
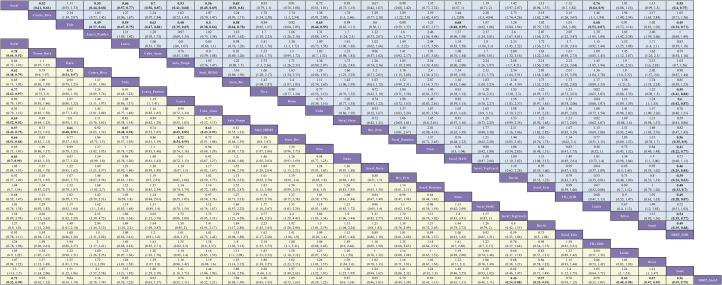
League table of pairwise comparisons from the Bayesian network meta-analysis for first-line immunotherapy and targeted regimens in advanced HCC (overall population). The lower, yellow-shaded triangle presents the HRs and 95% CIs for OS, while the upper, blue-shaded triangle presents the results for PFS. An HR less than 1.00 indicates a better survival benefit for the column-defining treatment compared to the row-defining treatment.

For OS in advanced HCC, five regimens significantly reduced the risk of death versus sorafenib: sintilimab plus bevacizumab (Sinti-IBI305) (HR = 0.57; 95% CI, 0.43-0.75), camrelizumab plus rivoceranib (Camre-Rivo) (HR = 0.62; 95% CI, 0.48-0.79), atezolizumab plus bevacizumab (Atezo-Bev) (HR = 0.66; 95% CI, 0.51-0.84), anlotinib plus penpulimab (Anlo-Penpu) (HR = 0.69; 95% CI, 0.52-0.92), and SBRT followed by sorafenib (SBRT-Soraf) (HR = 0.72; 95% CI, 0.52-0.99). Of these, the four immunotherapy-based combinations all demonstrated robust survival benefits, with the top three regimens (Sinti-IBI305, Camre-Rivo, and Atezo-Bev) showing a particularly pronounced advantage. In contrast, the benefit from SBRT-Soraf was borderline, with an upper confidence limit near 1.00, indicating a less robust magnitude of benefit (**Figure 4**).

Regarding PFS, dovitinib (Doviti) had the least favorable point estimate versus sorafenib (HR = 1.42; 95% CI, 0.97-2.07), indicating no evidence of delayed progression and suggesting a potential for harm. While most immunotherapy-based regimens did not significantly improve PFS relative to sorafenib, five specific combinations yielded statistically significant benefits: camrelizumab plus rivoceranib (Camre-Rivo) (HR = 0.52; 95% CI, 0.41-0.66), anlotinib plus penpulimab (Anlo-Penpu) (HR = 0.53; 95% CI, 0.41-0.68), lenvatinib plus pembrolizumab (Lenva-Pembro) (HR = 0.55; 95% CI, 0.44-0.68), SBRT followed by sorafenib (SBRT-Soraf) (HR = 0.55; 95% CI, 0.40-0.75), and sintilimab plus bevacizumab (Sinti-IBI305) (HR = 0.56; 95% CI, 0.45-0.69) (**Figure 4**).

**Figure 5:**
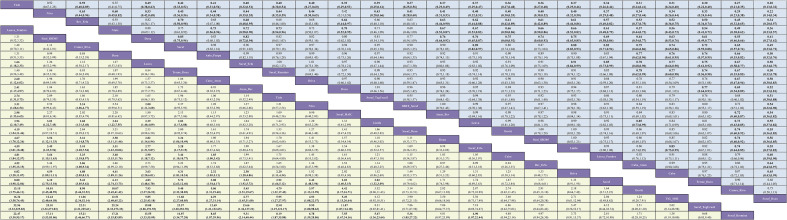
League table of pairwise comparisons from the Bayesian network meta-analysis for first-line immunotherapy and targeted regimens in advanced HCC (overall population). The lower, yellow-shaded triangle presents the results for ORR, and the upper, blue-shaded triangle presents the results for Grade ≥3 Adverse Events. Each cell provides the RR with its 95% CI for the comparison of the column-defining treatment versus the row-defining treatment. For ORR, an RR greater than 1.00 favors the column-defining treatment (indicating a higher response rate). For AEs ≥3, an RR less than 1.00 favors the column-defining treatment (indicating a better safety profile).

In the safety analysis, most regimens did not significantly increase toxicity compared to sorafenib. Notably, five treatments conferred a more favorable safety profile, with significantly lower rates of grade ≥3 AEs. These were predominantly ICI monotherapies or TKIs known for better tolerability: tislelizumab (Tisle) (RR = 0.42; 95% CI, 0.33-0.52), nivolumab (Nivo) (RR = 0.45; 95% CI, 0.36-0.56), bevacizumab plus erlotinib (Bev-Erlo) (RR = 0.70; 95% CI, 0.50-0.98), nintedanib (Ninte) (RR = 0.75; 95% CI, 0.61-0.92), and donafenib (Dona) (RR = 0.85; 95% CI, 0.75-0.96). Conversely, combination regimens involving traditional chemotherapy, such as sorafenib plus doxorubicin (Soraf-Doxo) (RR = 2.06; 95% CI, 1.39-3.04) and tigatuzumab plus sorafenib (TIG-SOR) (RR = 1.54; 95% CI, 1.36-1.75), resulted in a significantly higher risk of severe toxicity (**Figure 5**).

Regarding ORR, five regimens significantly increased the probability of tumor response versus sorafenib. The largest relative increase was observed with lenvatinib plus pembrolizumab (Lenva-Pembro) (RR = 8.00; 95% CI, 4.98-12.86), followed by durvalumab (Dura) (RR = 6.13; 95% CI, 2.74-13.73), sintilimab plus bevacizumab (Sinti-IBI305) (RR = 6.10; 95% CI, 2.75-13.54), tremelimumab plus durvalumab (Treme-Dura) (RR = 5.72; 95% CI, 2.63-12.46), and camrelizumab plus rivoceranib (Camre-Rivo) (RR = 5.42; 95% CI, 3.05-9.62). All differences were statistically significant (**Figure 5**).

#### 3.2.2 HBV/HCV infected subgroup

**Figure 6:**
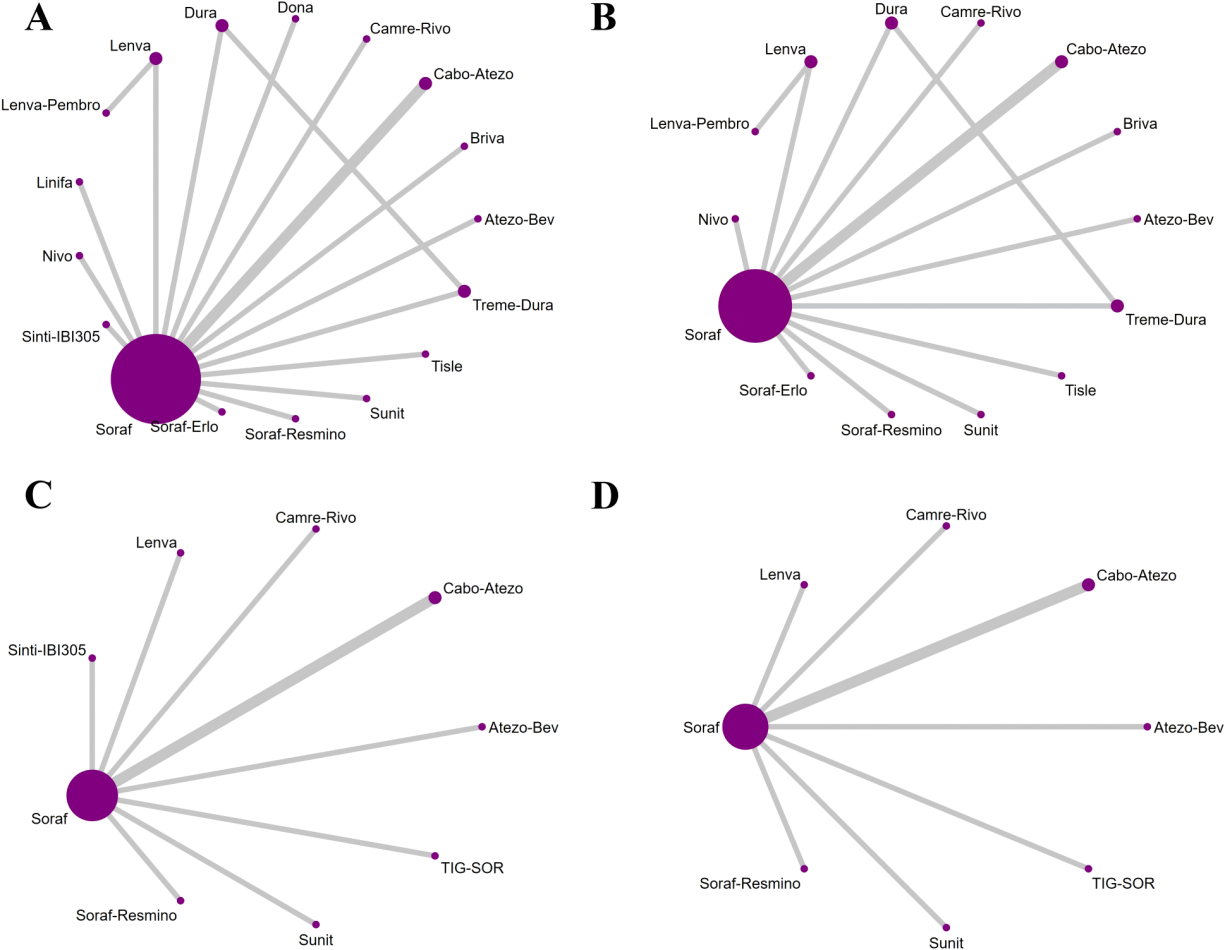
Network geometry of first-line immunotherapy and targeted regimens for OS and PFS in patient subgroups with HBV or HCV infection. The evidence networks correspond to the following: **(A)** OS in the HBV-positive subgroup; **(B)** PFS in the HBV-positive subgroup; **(C)** OS in the HCV-positive subgroup; and **(D)** PFS in the HCV-positive subgroup.

In the HBV-infected subgroup, the evidence network comprised 17 interventions for overall survival (OS) and 9 for progression-free survival (PFS); in the HCV-infected subgroup, the corresponding networks included 14 and 8 interventions, respectively (**Figure 6**).

**Figure 7:**
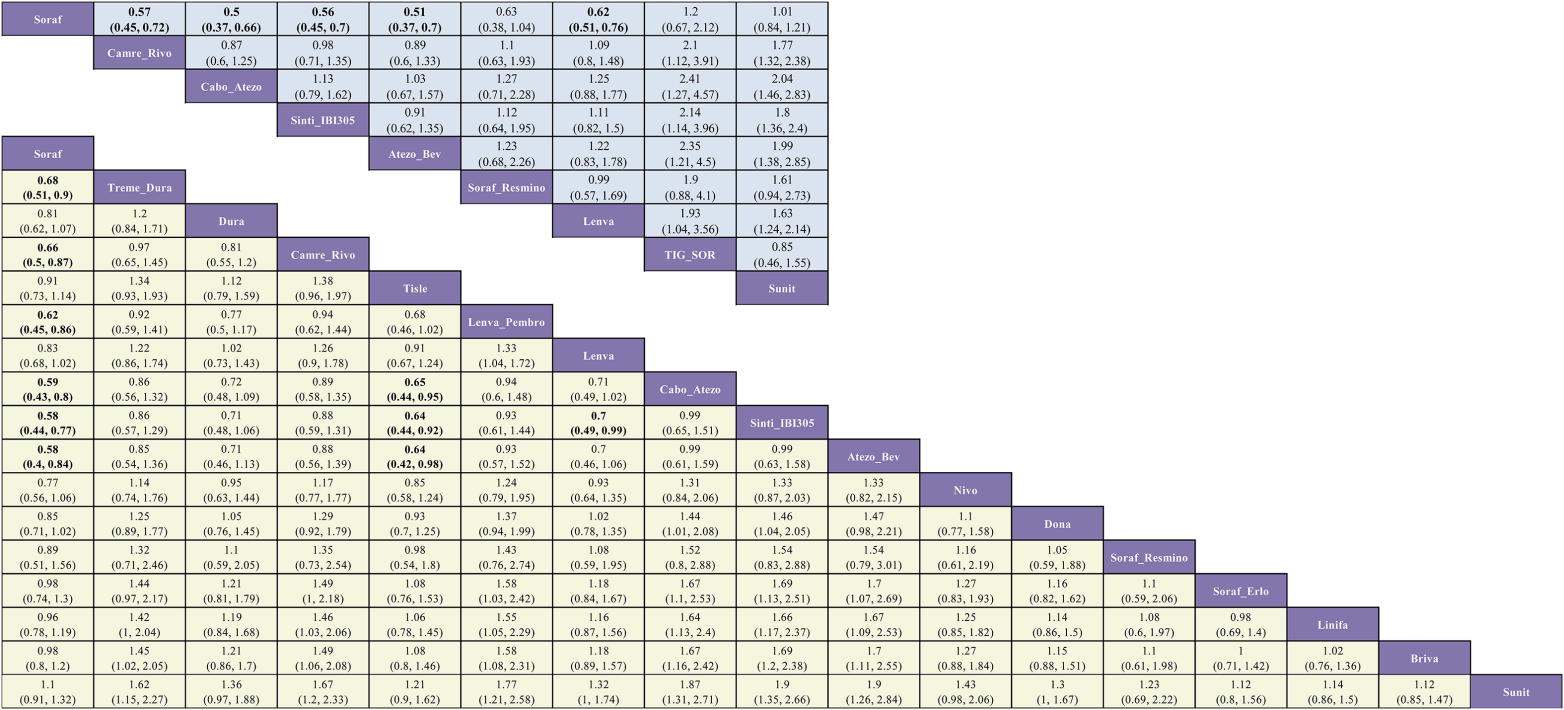
League table of pairwise comparisons from the Bayesian network meta-analysis for first-line immunotherapy and targeted regimens in the HBV-positive subgroup. The lower (yellow-shaded) and upper (blue-shaded) triangles display the results for OS and PFS, respectively. Each cell provides the HR with its 95% CI for the comparison of the column-defining treatment versus the row-defining treatment. An HR less than 1.00 indicates a better survival benefit for the column-defining treatment.

For OS in HBV-infected patients, five combination regimens significantly reduced mortality risk versus sorafenib: sintilimab plus bevacizumab (Sinti-IBI305) (HR = 0.58; 95% CI, 0.44-0.77), atezolizumab plus bevacizumab (Atezo-Bev) (HR = 0.58; 95% CI, 0.40-0.84), cabozantinib plus atezolizumab (Cabo-Atezo) (HR = 0.59; 95% CI, 0.43-0.80), lenvatinib plus pembrolizumab (Lenva-Pembro) (HR = 0.62; 95% CI, 0.45-0.86), and camrelizumab plus rivoceranib (Camre-Rivo) (HR = 0.66; 95% CI, 0.50-0.87) (**Figure 7**). In the hepatitis B virus (HBV)-infected subgroup, several regimens significantly prolonged PFS versus sorafenib: cabozantinib plus atezolizumab (Cabo-Atezo) (HR = 0.50; 95% CI, 0.37-0.66), atezolizumab plus bevacizumab (Atezo-Bev) (HR = 0.51; 95% CI, 0.37-0.70), sintilimab plus bevacizumab (Sinti-IBI305) (HR = 0.56; 95% CI, 0.45-0.70), camrelizumab plus rivoceranib (Camre-Rivo) (HR = 0.57; 95% CI, 0.45-0.72), and lenvatinib (Lenva) (HR = 0.62; 95% CI, 0.51-0.76) (**Figure 7**).

**Figure 8:**
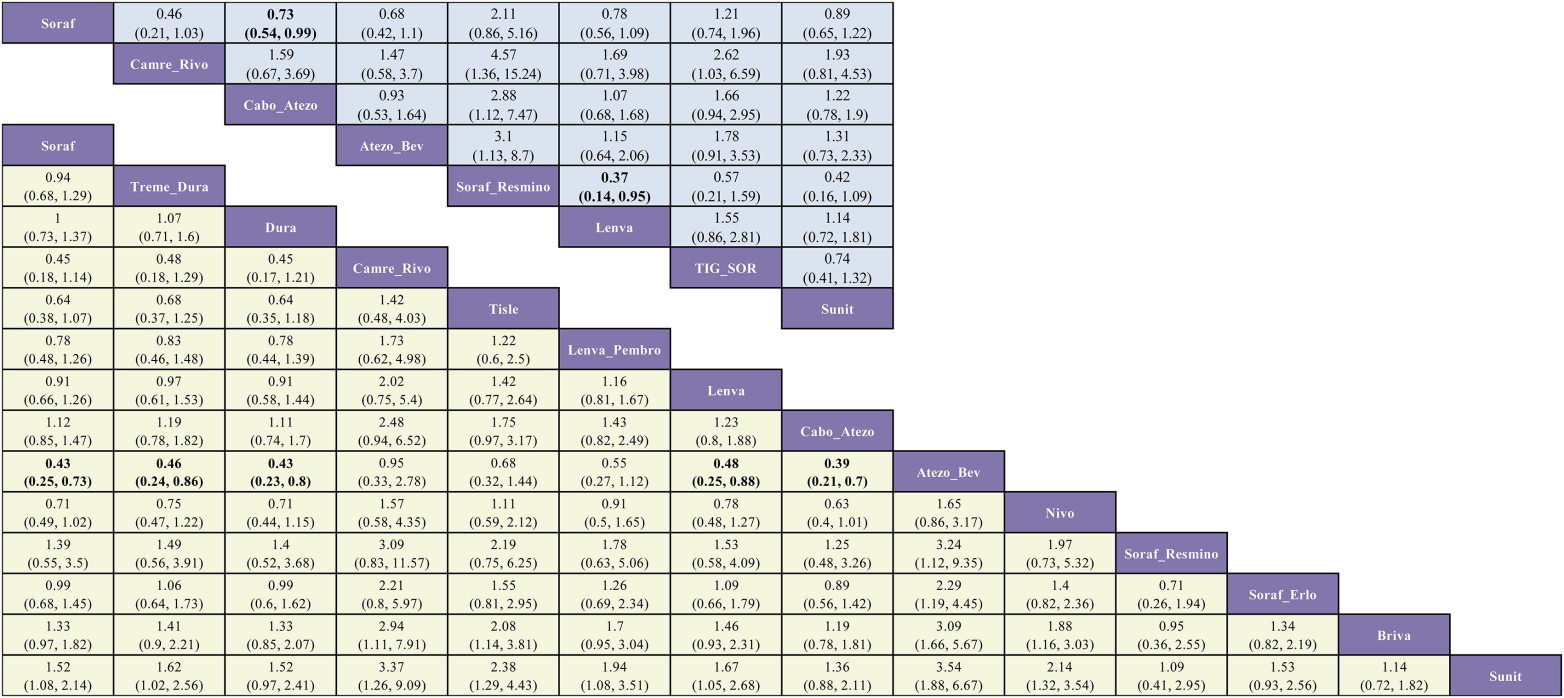
League table of pairwise comparisons from the Bayesian network meta-analysis for first-line immunotherapy and targeted regimens in the HCV-positive subgroup. The lower (yellow-shaded) and upper (blue-shaded) triangles display the results for OS and PFS, respectively. Each cell provides the HR with its 95% CI for the comparison of the column-defining treatment versus the row-defining treatment. An HR less than 1.00 indicates a better survival benefit for the column-defining treatment.

Among patients with hepatitis C virus (HCV)-associated HCC, most regimens did not improve overall survival (OS) relative to sorafenib. Atezo-Bev was the only regimen to confer a significant OS advantage (HR = 0.43; 95% CI, 0.25-0.73), whereas sunitinib (Sunit) was inferior (HR = 1.52; 95% CI, 1.08-2.14). In the corresponding PFS analysis, Cabo-Atezo achieved the sole statistically significant benefit (HR = 0.73; 95% CI, 0.54-0.99). Although Camre-Rivo (HR = 0.46; 95% CI, 0.21-1.03) yielded the most favorable point estimate, and Atezo-Bev (HR = 0.68; 95% CI, 0.42-1.10), Lenva (HR = 0.78; 95% CI, 0.56-1.09), and Sunit (HR = 0.89; 95% CI, 0.65-1.22) were numerically favorable, these differences did not reach statistical significance (**Figure 8**).

#### 3.2.3 Non-viral infected subgroup

**Figure 9:**
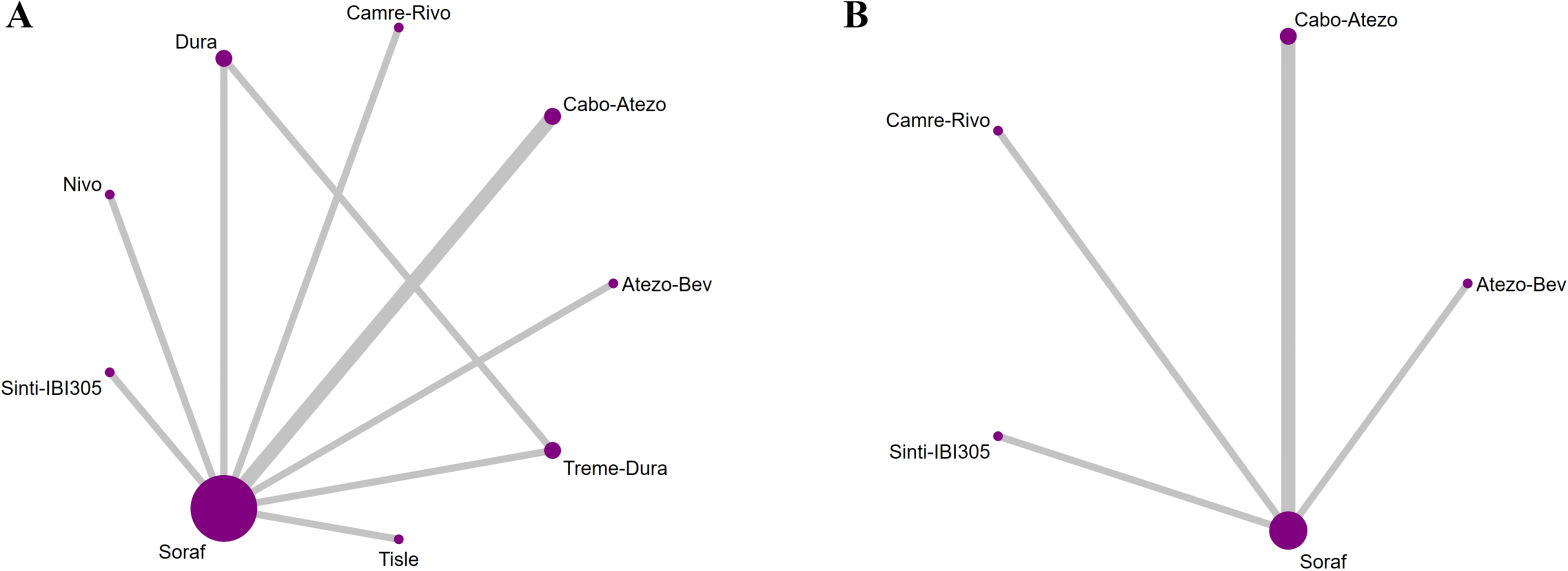
Network geometry of first-line immunotherapy and targeted regimens for advanced HCC in the non-viral subgroup. The evidence networks correspond to the outcomes of **(A)** OS and **(B)** PFS.

In the subgroup with non-viral etiology, the evidence network comprised nine interventions for OS and five for PFS. (**Figure 9**).

**Figure 10:**
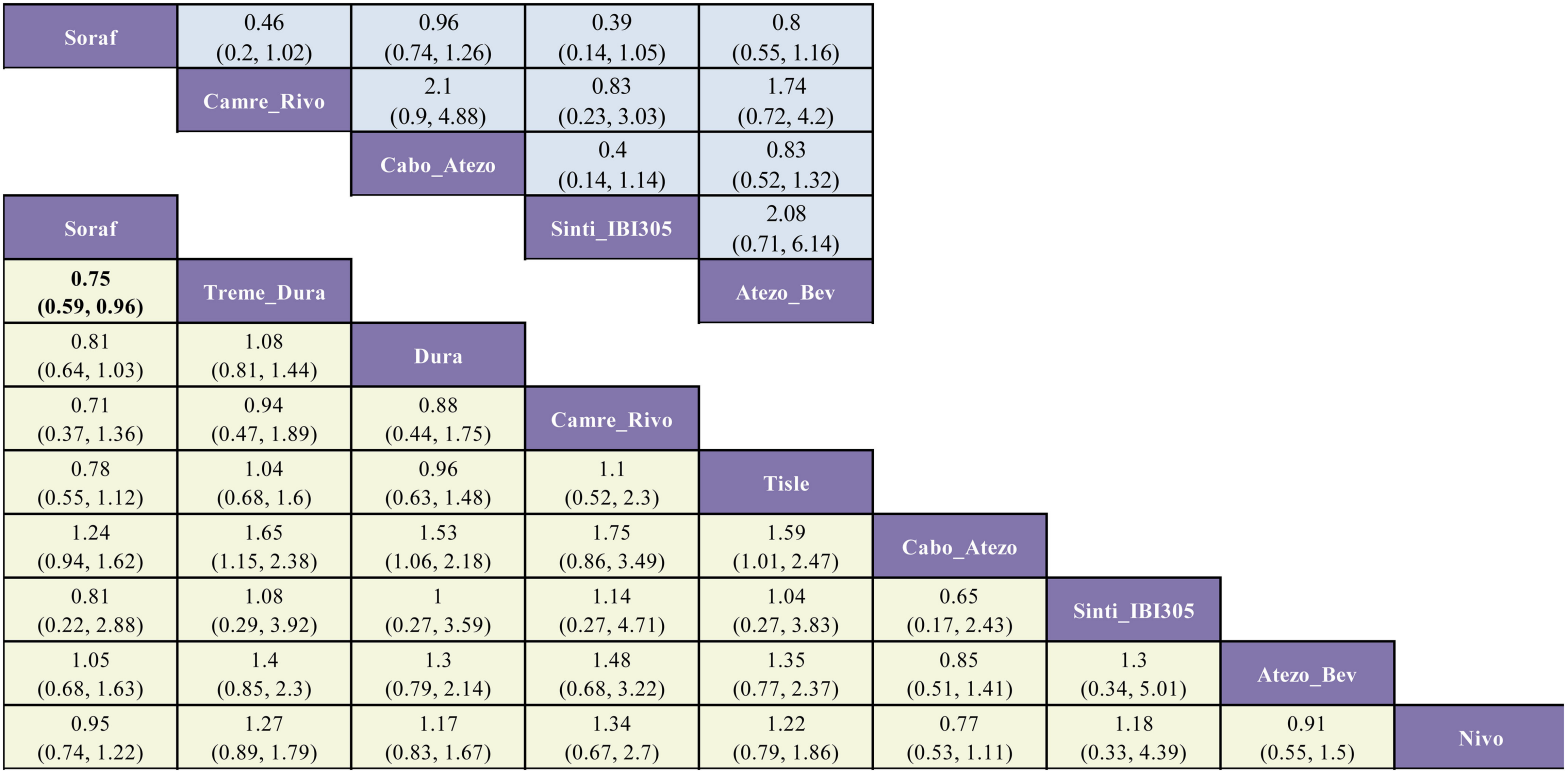
League table of pairwise comparisons from the Bayesian network meta-analysis for first-line immunotherapy and targeted regimens in the non-viral subgroup. The lower (yellow-shaded) and upper (blue-shaded) triangles display the results for OS and PFS, respectively. Each cell provides the HR with its 95% CI for the comparison of the column-defining treatment versus the row-defining treatment. An HR less than 1.00 indicates a better survival benefit for the column-defining treatment.

In the OS analysis, most regimens did not improve survival versus sorafenib. Only tremelimumab plus durvalumab (Treme-Dura) achieved a statistically significant benefit (HR = 0.75; 95% CI, 0.59-0.96). Camrelizumab plus rivoceranib (Camre-Rivo) (HR = 0.71; 95% CI, 0.37-1.36), tislelizumab (Tisle) (HR = 0.78; 95% CI, 0.55-1.12), and durvalumab (Dura) (HR = 0.81; 95% CI, 0.64-1.03) were numerically favorable (HR < 1.0) but not statistically significant. In the corresponding PFS analysis, no regimen demonstrated a significant advantage over sorafenib. Sintilimab plus bevacizumab (Sinti-IBI305) (HR = 0.39; 95% CI, 0.14-1.05) and Camre-Rivo (HR = 0.46; 95% CI, 0.20-1.02) yielded the most favorable point estimates but remained nonsignificant, whereas cabozantinib plus atezolizumab (Cabo-Atezo) (HR = 0.96; 95% CI, 0.74-1.26) showed no apparent improvement (**Figure 10**).

### 3.3 Ranking analysis

Bayesian ranking (SUCRA) results(**Supplementary Figures S1**-**S10**). In the overall population, Sinti-IBI305 had the highest probability of ranking first for OS (93.4%), followed by Camre-Rivo (91.4%). Rankings varied by viral etiology. Among patients with HBV infection, Sinti-IBI305 ranked first for OS (SUCRA, 92.1%), with Camre-Rivo second (82.3%). In the HCV subgroup, Atezo-Bev ranked first (92.7%), followed by Camre-Rivo (85.4%). In patients without viral infection, Treme-Dura and Camre-Rivo ranked first and second for OS (90.4% and 70.0%, respectively).

For PFS, Atezo-Bev ranked first in the overall population (93.3%), followed by Lenva-Pembro (88.1%). Within the HBV subgroup, Atezo-Bev ranked first for PFS (91.7%), with Camre-Rivo second (85.0%). In HCV-infected patients, Camre-Rivo (96.0%) and Cabo-Atezo (79.8%) were the top-ranked regimens. For patients without viral infection, Sinti-IBI305 (85.7%) and Camre-Rivo (77.1%) ranked first and second, respectively.

Regarding safety (incidence of adverse events of grade ≥3), tislelizumab had the highest probability of being the safest regimen (98.7%), followed by nivolumab (96.8%). For ORR, Lenva-Pembro (93.9%) and Sinti-IBI305 (91.3%) were the two highest-ranked regimens.

### 3.4 Convergence, consistency, sensitivity, and publication bias

Model convergence was confirmed by visual diagnostics and the Gelman–Rubin statistic, indicating well-mixed, stable MCMC sampling across chains (**Supplementary Figures 15**–**34**). To evaluate the network coherence assumption, we contrasted DIC values from consistency and inconsistency specifications; for all endpoints, the DIC differences were <5 (**Supplementary Table S3**), providing no evidence of meaningful global inconsistency and supporting exchangeability between direct and indirect estimates. As a sensitivity analysis, we additionally compared model fit between fixed-effect and random-effects specifications by calculating their DIC values. Across all endpoints, the DIC differences were <5, indicating that the primary findings were robust to the choice of effect model. We further assessed the robustness of the HBV-, HCV-, and non-infected subgroups using a leave-one-out approach, sequentially excluding each study to examine whether the estimated effects and statistical significance of each treatment versus sorafenib materially changed. The results remained consistent across iterations, supporting the stability of the subgroup findings. Potential small-study and publication biases were examined with funnel plots for OS, PFS, ORR, and grade ≥3 adverse events; the plots were broadly symmetrical without conspicuous outliers, suggesting a low likelihood of small-study effects or publication bias (**Supplementary Figures 11**–**14**).

## 4 Discussion

As the sixth most common malignancy and the third leading cause of cancer-related death worldwide (1), HCC requires continual optimization of therapeutic strategies to improve outcomes. The advent of immune checkpoint inhibitors and next-generation targeted agents has reshaped the first-line treatment landscape for advanced HCC. Yet most pivotal trials employed sorafenib as the control arm, leaving few direct head-to-head comparisons among contemporary immunotherapy-based or targeted combinations. In addition, the modifying influence of viral etiology has often been underappreciated. Against this backdrop, our comprehensive network meta-analysis systematically compared current first-line regimens and, to our knowledge, provides the first stratified synthesis across HBV, HCV, and non-viral subgroups, with the aim of furnishing an evidence-based framework for more precise, individualized care.

In the overall population, several regimens outperformed sorafenib. For OS, Sinti-IBI305 conferred the greatest benefit (HR, 0.57; 95% CI, 0.43–0.75), followed by Camre-Rivo (HR, 0.62; 95% CI, 0.48–0.79) and Atezo-Bev (HR, 0.66; 95% CI, 0.51–0.84). For PFS, Camre-Rivo achieved the best outcome (HR, 0.52; 95% CI, 0.41–0.66), closely followed by Anlo-Penpu (HR, 0.53; 95% CI, 0.41–0.68). Regarding ORR, Lenva-Pembro yielded the highest response (RR, 8.00; 95% CI, 4.98–12.86). From a safety perspective, tislelizumab and nivolumab were associated with the lowest rates of grade ≥3 adverse events. The superior safety profile observed with tislelizumab and nivolumab monotherapy is likely attributable to the absence of additive toxicities associated with combination partners. Regimens pairing ICIs with anti-angiogenic agents or TKIs introduce distinct class-specific adverse events—such as hypertension and bleeding risks from VEGF inhibition, or diarrhea and hand-foot skin reactions from multi-kinase inhibitors—which contribute to higher overall rates of severe toxicity (6, 21). By contrast, PD-1 inhibitors as monotherapy possess a more focused mechanism of action. Although they carry a risk of immune-related adverse events, phase III trials such as CheckMate 459 and RATIONALE-301 have consistently demonstrated that severe grade ≥3 toxicities are less frequent with PD-1 blockade compared to the cumulative systemic toxicities inherent to continuous TKI exposure like sorafenib (11, 15). Taken together, these data highlight meaningful efficacy gains with several ICI-based combinations, whereas single-agent PD-1 inhibitors may offer advantageous tolerability profiles, a consideration that can inform regimen selection in routine practice.

The subgroup analyses by viral etiology revealed distinct efficacy patterns. In patients with HBV infection, Sinti-IBI305 (HR = 0.58; 95% CI, 0.44-0.77) and Atezo-Bev (HR = 0.58; 95% CI, 0.40-0.84) were the top performers for OS, closely followed by Cabo-Atezo (HR = 0.59; 95% CI, 0.43-0.80). For PFS in this subgroup, Cabo-Atezo (HR = 0.50; 95% CI, 0.37-0.66) and Atezo-Bev (HR = 0.51; 95% CI, 0.37-0.70) conferred the greatest benefit. Among patients with HCV-associated HCC, Atezo-Bev was the only regimen to improve OS significantly (HR = 0.43; 95% CI, 0.25-0.73), and Cabo-Atezo was the only regimen to achieve a statistically significant PFS advantage (HR = 0.73; 95% CI, 0.54-0.99). In non-viral HCC, the STRIDE regimen (tremelimumab plus durvalumab) was the sole therapy associated with a significant OS benefit (HR = 0.75; 95% CI, 0.59-0.96).

The superior performance of several PD-1/PD-L1–based combinations in HBV-infected patients, such as Sinti-IBI305, Atezo-Bev, and Cabo-Atezo, is biologically plausible. Chronic HBV infection provides persistent inflammatory and antigenic stimulation that drives intrahepatic T cells toward an exhausted, PD-1-high phenotype with impaired effector function (3). Accordingly, HBV-associated tumors often harbor abundant PD-1–positive tumor-infiltrating lymphocytes (TILs) and exhibit features of a “hot” immune microenvironment with higher PD-L1 expression, characteristics that are associated with aggressive disease (e.g., portal vein tumor thrombus) yet render tumors more susceptible to PD-(L)1 blockade (30). Notably, each of the above regimens incorporates VEGF-pathway inhibition. Beyond promoting vascular normalization and improving perfusion, VEGF blockade diminishes immunosuppressive cell populations (Tregs, MDSCs, TAMs) and supports dendritic-cell maturation and cytotoxic T-cell activation, thereby relieving local immunosuppression and potentiating checkpoint inhibition (3, 30). The convergence of these mechanisms, namely targeting HBV-driven T-cell exhaustion while simultaneously lifting VEGF-mediated immune suppression, provides a coherent rationale for the observed survival advantages of these combinations in HBV-associated HCC.

By contrast, only Atezo-Bev conferred a significant OS advantage in patients with HCV infection. Mechanistically, the HCV core protein upregulates VEGF via pathways including HIF-1α, STAT3-AR, and AP-1 (31). Consistent histologic and functional data show that HCV-HCC harbors higher VEGF expression and micro vessel density, and that VEGF inhibition markedly attenuates its pro-angiogenic activity (32). VEGF also orchestrates immune evasion by suppressing dendritic-cell maturation and antigen presentation, mobilizing or expanding Tregs, TAMs, and MDSCs, and restricting CD8^+^ T-cell infiltration through aberrant vasculature (33). Anti-VEGF therapy can induce vascular normalization, alleviate local immunosuppression, and reprogram the tumor microenvironment from inhibitory to immune-permissive states (33); subsequent PD-1/PD-L1 blockade then restores and amplifies T-cell cytotoxicity. In an etiologic context dominated by VEGF-driven immunosuppression, Atezo-Bev simultaneously dismantles vascular and immunologic barriers, providing a coherent explanation for its unique survival benefit in the HCV subgroup.

In the non-viral subgroup, the unique survival benefit observed with the STRIDE regimen suggests that dual checkpoint blockade may address specific resistance mechanisms inherent to this etiology. Unlike viral HCC, which is typically characterized by a ‘hot’ microenvironment responsive to PD-1 blockade, non-viral HCC, particularly cases driven by non-alcoholic steatohepatitis (NASH), exhibits a distinct immunopathology (34). Pfister et al. demonstrated that NASH-associated aberrant T-cell activation impairs tumor surveillance, a dysfunction that may not be reversed by PD-1 blockade alone and could potentially be exacerbated by immune-mediated tissue damage (34). Mechanistically, the inclusion of tremelimumab (anti-CTLA-4) in the STRIDE regimen is critical. While PD-L1 inhibitors act primarily at the effector phase within the tumor, CTLA-4 inhibition operates upstream during the priming phase in lymph nodes, promoting T-cell proliferation and diversifying the peripheral T-cell repertoire (5, 35). This ‘priming’ effect may be essential in non-viral HCC to recruit novel, high-avidity T-cell clones unaffected by the NASH metabolic environment, thereby overcoming the resistance often observed with anti-PD-L1 monotherapy in this population (3, 5).

While our mechanistic discussion primarily centers on the established PD-(L)1 and VEGF pathways, it is increasingly recognized that the regulation of the etiology-specific immune microenvironment involves more complex cell death modalities. Emerging literature has highlighted the role of PANoptosis, a unique inflammatory programmed cell death pathway involving the interplay of pyroptosis, apoptosis, and necroptosis, as a novel frontier in HCC biology. As proposed by Xiang et al. (36), PANoptosis modulation provides a cutting-edge molecular perspective on how virus-related HCCs shape their immune landscape. Correlating existing pathway inhibition mechanisms with such novel regulatory nodes could further enrich the scientific understanding of etiology-stratified therapy and point toward potential synergistic targets for next-generation regimens.

Our results generally accord with the meta-analyses by She et al. (37) and Zhang et al. (37, 38), which established the broad superiority of PD-(L)1 inhibitor-based combinations over sorafenib regarding OS and PFS. However, our study diverges in the precision of estimates for newer targeted immunotherapy combinations. While Zhang et al. constructed their network relying solely on sorafenib as the common comparator (38–40), our analysis incorporated lenvatinib as a second connector node. This topological difference is not merely methodological; it fundamentally alters the result stability for lenvatinib-based regimens. By effectively integrating direct evidence from the LEAP-002 trial, which compared lenvatinib plus pembrolizumab with Lenvatinib (19), our network reduces the statistical uncertainty inherent in long indirect comparison chains, such as the sequence linking lenvatinib plus pembrolizumab to sorafenib through lenvatinib. This structural advantage likely explains why we were able to identify lenvatinib plus pembrolizumab as the definitive top-ranked regimen for ORR (RR = 8.00) with narrower credible intervals compared to prior networks that lacked this direct linkage.

This Bayesian network meta-analysis yielded several clinically salient observations. First, etiologic stratification provides quantitative support for individualized therapy. In the HCV-infected subgroup, only Atezo-Bev achieved a significant OS benefit (HR = 0.43; 95% CI, 0.25-0.73), whereas for PFS only Cabo-Atezo reached significance (HR = 0.73; 95% CI, 0.54-0.99). These findings indicate relatively limited options in HCV-associated HCC and offer clear therapeutic guidance for this population. In non-viral HCC, the STRIDE regimen (durvalumab plus tremelimumab) was the sole strategy to confer a significant OS advantage (HR = 0.75; 95% CI, 0.59-0.96), suggesting that dual-checkpoint blockade may hold a context-specific benefit. Second, by jointly evaluating efficacy and safety, our analysis enables a balanced efficacy-toxicity appraisal. SUCRA-based rankings delineated leading options across OS and PFS and identified optimal choices for ORR and grade ≥3 AEs. Notably, tislelizumab and nivolumab exhibited outstanding safety profiles (SUCRA 98.7% and 96.8%, respectively), while Lenva-Pembro ranked highest for ORR (RR = 8.00; 95% CI, 4.98-12.86). This multidimensional evidence base can assist clinicians in selecting regimens that align with individual patient priorities and tolerability, thereby supporting more precise, personalized first-line treatment.

Furthermore, it is crucial to acknowledge the boundaries of clinical applicability regarding special populations, particularly patients with impaired liver function (Child-Pugh class B) and those with macrovascular invasion (e.g., portal vein tumor thrombosis [PVTT]). The majority of phase III RCTs underpinning our network, such as IMbrave150, HIMALAYA, LEAP-002, and CARES-310, restricted enrollment to patients with Child-Pugh class A liver function to minimize competing risks of hepatic failure (6, 7, 16, 19). Consequently, the efficacy and safety hierarchies established in our analysis may not fully extrapolate to Child-Pugh class B patients, a subgroup historically prone to lower tolerance for systemic therapies and higher rates of decompensation. Similarly, while regimens containing potent anti-angiogenic components (e.g., lenvatinib or bevacizumab-based combinations) have demonstrated efficacy in patients with high tumor burden or PVTT in individual trials (6, 21), our network meta-analysis did not specifically stratify by vascular invasion status due to variable reporting across studies. Thus, while our findings provide a robust framework for the standard-risk population, treatment decisions for these high-risk phenotypes require careful, individualized assessment, often necessitating reliance on real-world evidence to complement trial data.

Despite offering clinically relevant insights, this study has limitations. First, several regimens in the network were informed by a single randomized trial (for example, lenvatinib plus pembrolizumab [LEAP-002] (19), cabozantinib plus atezolizumab [COSMIC-312] (8), and the STRIDE regimen [HIMALAYA] (7)), which increases uncertainty around effect estimates and SUCRA-based rankings and may unbalance the network. Second, clinical heterogeneity across trials, including differences in eligibility criteria, geographic distribution, and follow-up duration, could affect the precision of treatment effects. Notably, the presence and extent of macrovascular invasion constitute a significant source of heterogeneity often under-addressed in aggregate analyses. As highlighted by Wang et al. (41), specific high-risk subtypes, such as HCC complicated with tumor thrombus in the hepatic vein, inferior vena cava, or right atrium, exhibit distinct therapeutic responses and safety profiles compared to ordinary advanced HCC. The varying proportions of these complex thrombus phenotypes across the included RCTs likely contributed to the observed heterogeneity in our network; however, due to the lack of granular data reporting in primary studies, we were unable to perform a stratified analysis to isolate this specific confounder. Third, for regimens evaluated in trials without a sorafenib comparator, relative effects rely primarily on indirect comparisons; although this approach is standard in network meta-analysis, such inferences are inherently less secure than direct, head-to-head evidence.

However, a limitation remains regarding the granularity of subgroup reporting. While our overall network analysis incorporated a substantial dataset of 24 RCTs involving 13,572 participants, not all primary studies provided survival outcomes stratified by viral etiology. Consequently, specific interventions within the etiology-based subnetworks are informed by a reduced number of studies compared to the overall population. Nevertheless, it is important to emphasize that this analysis represents the totality of currently available evidence. By conducting an exhaustive search across four major databases and integrating all eligible direct and indirect comparisons, we have maximized the statistical power attainable given the current state of the literature. Therefore, despite the inherent reporting gaps in primary trials, these findings reflect the most robust and comprehensive synthesis possible at present, offering valid and actionable insights for clinical decision-making.

Several priorities for future research emerge from these findings. First, adequately powered head-to-head trials should compare top-ranked combinations, such as atezolizumab plus bevacizumab versus cabozantinib plus atezolizumab, to provide definitive comparative-effectiveness data for first-line selection. Second, studies should evaluate treatment sequencing and conversion strategies, including optimal second-line options after failure of specific first-line regimens and the ordering of immunotherapy and targeted therapy in routine care. In particular, as immunotherapy-based combinations become the dominant first-line standard, exploring the potential of immune checkpoint inhibitor (ICI) rechallenge is becoming increasingly critical. Recent reviews suggest that ICI rechallenge may serve as a key subsequent strategy for patients who experience disease progression, thereby contributing to a more systematic ‘full-course’ treatment management framework (42). Third, high-quality real-world evidence (for example, prospective registries and pragmatic trials) is needed to complement RCTs, enhance external validity, and inform care in under-represented populations (such as older adults and patients with impaired liver function), where safety and effectiveness in routine practice may diverge from trial results.

## Data Availability

The original contributions presented in the study are included in the article/**Supplementary Material**, further inquiries can be directed to the corresponding author/s.
