# Supplementary material for: Efficacy and safety of first-line immunotherapy and targeted therapy in advanced HCC: a network meta-analysis with subgroup analysis based on HBV and HCV infection
Source: Front Immunol. 2026 Jan 29;17:1706446. doi: 10.3389/fimmu.2026.1706446 (PMC12894325; doi:10.3389/fimmu.2026.1706446)
Supplement: Supplementary file 1 [file DataSheet1.pdf]

Efficacy and safety of first-line immunotherapy and targeted therapy in advanced HCC: a network meta-analysis with subgroup analysis based on HBV and HCV infection.

Table of Contents

Title	Content	page
Table S1	PRISMA NMA Checklist of Items to Include When Reporting a Systematic Review Involving a Network Meta-analysis	3-6
Table S2	Literature Search Strategy	6-8
Table S3	Consistency and Inconsistency Model Fit Comparison and Heterogeneity Assessment Across Endpoints in the Bayesian Network Meta-Analysis	8
Table S4	Fixed-Effect vs Random-Effects Model Fit Comparison and Heterogeneity Assessment Across Endpoints in the Bayesian Network Meta-Analysis	9
Table S5	Tumor Response Assessment Criteria and Baseline Liver Reserve Function (Across Trials)	10
Table S6	Etiology Subgroups (Experimental vs Control)	13
Figure S1	Rank Probability Heatmap of Treatment Regimens for OS in Advanced HCC.	14
Figure S2	Rank Probability Heatmap of Treatment Regimens for PFS in Advanced HCC.	14
Figure S3	Rank Probability Heatmap of Treatment Regimens for ORR in Advanced HCC.	15
Figure S4	Rank Probability Heatmap of Treatment Regimens for AE≥3 in Advanced HCC.	15
Figure S5	Rank Probability Heatmap of Treatment Regimens for OS in HBV-positive Advanced HCC.	15
Figure S6	Rank Probability Heatmap of Treatment Regimens for OS in HCV-positive Advanced HCC.	16
Figure S7	Rank Probability Heatmap of Treatment Regimens for OS in NBNCAdvanced HCC.	17
Figure S8	Rank Probability Heatmap of Treatment Regimens for PFS in HBV-positive Advanced HCC.	18
Figure S9	Rank Probability Heatmap of Treatment Regimens for PFS in HCV-positive Advanced HCC.	19
Figure S10	Rank Probability Heatmap of Treatment Regimens for PFS in NBNC Advanced HCC.	20
Figure S11	Comparison adjusted funnel plot for overall survival in first line therapie	21
Figure S12	Comparison adjusted funnel plot for progression free survival in first line therapies	22
Figure S13	Comparison adjusted funnel plot for objective response rate in first line therapies	23
Figure S14	Comparison adjusted funnel plot for grade ≥3 adverse events in first line therapies	24
Figure S15	MCMC trace and posterior density plots for OS in advanced hepatocellular carcinoma	25
Figure S16	MCMC trace and posterior density plots for PFS in advanced hepatocellular carcinoma	26
Figure S17	MCMC trace and posterior density plots for ORR in advanced hepatocellular carcinoma	27
Figure S18	MCMC trace and posterior density plots for AEs≥3 in advanced hepatocellular carcinoma	28
Figure S19	MCMC trace and posterior density plots for OS in HBV-positive advanced hepatocellular carcinoma	29
Figure S20	MCMC trace and posterior density plots for OS in HCV-positive advanced hepatocellular carcinoma	30
Figure S21	MCMC trace and posterior density plots for OS in NBNC advanced hepatocellular carcinoma	31
Figure S22	MCMC trace and posterior density plots for PFS in HBV-positive advanced hepatocellular carcinoma	32
Figure S23	MCMC trace and posterior density plots for PFS in HCV-positive advanced hepatocellular carcinoma	33
Figure S24	MCMC trace and posterior density plots for PFS in NBNC advanced hepatocellular carcinoma	34
Figure S25	Convergence diagnostics for OS in advanced hepatocellular carcinoma	35
Figure S26	Convergence diagnostics for PFS in advanced hepatocellular carcinoma	36

Figure S27	Convergence diagnostics for ORR in advanced hepatocellular carcinoma	37
Figure S28	Convergence diagnostics for AEs≥3 in advanced hepatocellular carcinoma	38
Figure S29	Convergence diagnostics for OS in HBV-positive advanced hepatocellular carcinoma	39
Figure S30	Convergence diagnostics for OS in HCV-positive advanced hepatocellular carcinoma	40
Figure S31	Convergence diagnostics for OS in NBNC advanced hepatocellular carcinoma	41
Figure S32	Convergence diagnostics for PFS in HBV-positive advanced hepatocellular carcinoma	42
Figure S33	Convergence diagnostics for PFS in HCV-positive advanced hepatocellular carcinoma	43
Figure S34	Convergence diagnostics for PFS in NBNC advanced hepatocellular carcinoma	44

Table S1 PRISMA NMA Checklist of Items to Include When Reporting a Systematic Review Involving a Network Meta-analysis

Section/Topic	Item #	Checklist Item	Reported on Page #
**TITLE**			
Title	1	Identify the report as a systematic review *incorporating anetwork meta-analysis (or related form of meta-analysis).*	**1**
**ABSTRACT**			
Structured summary	2	Provide a structured summary including, as applicable:<br>**Background:** main objectives<br>**Methods:** data sources; study eligibility criteria, participants, and interventions; study appraisal; and *synthesis methods, such as network meta-analysis.*<br>**Results:** number of studies and participants identified; summary estimates with corresponding confidence/credible intervals; *treatment rankings may also be discussed. Authors may choose to summarize pairwise comparisons against a chosen treatment included in their analyses for brevity.*<br>**Discussion/Conclusions:** limitations; conclusions and implications of findings.<br>**Other:** systematic review registration number with registry name.	1
**INTRODUCTION**			
Rationale	3	Describe the rationale for the review in the context of what is already known, *including mention of why a network meta-analysis has been conducted.*_	**1**
Objectives	4	Provide an explicit statement of questions being addressed, with reference to participants, interventions, comparisons, outcomes, and study design (PICOS).	1-2
**METHODS**			
Protocol and registration	5	Indicate whether a review protocol exists and if and where it can be accessed (e.g., Web address); and, if available, provide registration information, including registration number.	2
Eligibility criteria	6	Specify study characteristics (e.g., PICOS, length of follow-up) and report characteristics (e.g., years considered, language, publication status) used as criteria for eligibility, giving rationale. *Clearly describe eligible treatments included in the treatment network, and note whether any have been clustered or merged into the same node (with justification).*_	**2**
Information sources	7	Describe all information sources (e.g., databases with dates of coverage, contact with study authors to identify additional studies) in the search and date	2

		last searched.	
Search	8	Present full electronic search strategy for at least one database, including any limits used, such that it could be repeated.	2，Supplementary TableS2
Study selection	9	State the process for selecting studies (i.e., screening, eligibility, included in systematic review, and, if applicable, included in the meta-analysis).	**2-3**
Data collection process	10	Describe method of data extraction from reports (e.g., piloted forms, independently, in duplicate) and any processes for obtaining and confirming data from investigators.	3
Data items	11	List and define all variables for which data were sought (e.g., PICOS, funding sources) and any assumptions and simplifications made.	3
**Geometry of the network**	**S1**	Describe methods used to explore the geometry of the treatment network under study and potential biases related to it. This should include how the evidence base has been graphically summarized for presentation, and what characteristics were compiled and used to describe the evidence base to readers.	**2-3**
Risk of bias within individual studies	12	Describe methods used for assessing risk of bias of individual studies (including specification of whether this was done at the study or outcome level), and how this information is to be used in any data synthesis.	3
Summary measures	13	State the principal summary measures (e.g., risk ratio, difference in means). *Also describe the use of additional summary measures assessed, such as treatment rankings and surface under the cumulative ranking curve (SUCRA) values, as well as modified approaches used to present summary findings from meta-analyses.*	3
Planned methods of analysis	14	Describe the methods of handling data and combining results of studies for each network meta-analysis. This should include, but not be limited to:<br>• *Handling of multi-arm trials;*<br>• *Selection of variance structure;*<br>• *Selection of prior distributions in Bayesian analyses; and*<br>• *Assessment of model fit.*	3
**Assessment of Inconsistency**	**S2**	Describe the statistical methods used to evaluate the agreement of direct and indirect evidence in the treatment network(s) studied. Describe efforts taken to address its presence when found.	3
Risk of bias across studies	15	Specify any assessment of risk of bias that may affect the cumulative evidence (e.g., publication bias, selective reporting within studies).	**3**
Additional analyses	16	Describe methods of additional analyses if done, indicating which were pre-specified. This may include, but not be limited to, the following:<br>• Sensitivity or subgroup analyses;<br>• Meta-regression analyses;<br>• *Alternative formulations of the treatment network; and*<br>• *Use of alternative prior distributions for Bayesian analyses (if applicable).*_	**3**

RESULTS†

Study selection	17	Give numbers of studies screened, assessed for eligibility, and included in the review, with reasons for exclusions at each stage, ideally with a flow diagram.	4
**Presentation of network structure**	**S3**	Provide a network graph of the included studies to enable visualization of the geometry of the treatment network.	11,15
**Summary of network geometry**	**S4**	Provide a brief overview of characteristics of the treatment network. This may include commentary on the abundance of trials and randomized patients for the different interventions and pairwise comparisons in the network, gaps of evidence in the treatment network, and potential biases reflected by the network structure.	13-17
Study characteristics	18	For each study, present characteristics for which data were extracted (e.g., study size, PICOS, follow-up period) and provide the citations.	5-9,Table 1-2
Risk of bias within studies	19	Present data on risk of bias of each study and, if available, any outcome level assessment.	10,Figure 2
Results of individual studies	20	For all outcomes considered (benefits or harms), present, for each study: 1) simple summary data for each intervention group, and 2) effect estimates and confidence intervals. *Modified approaches may be needed to deal with information from larger networks.*	11-18
Synthesis of results	21	Present results of each meta-analysis done, including confidence/credible intervals. *In larger networks, authors may focus on comparisons versus a particular comparator (e.g. placebo or standard care), with full findings presented in an appendix. League tables and forest plots may be considered to summarize pairwise comparisons.* If additional summary measures were explored (such as treatment rankings), these should also be presented.	11-19
**Exploration for inconsistency**	**S5**	Describe results from investigations of inconsistency. This may include such information as measures of model fit to compare consistency and inconsistency models, *P* values from statistical tests, or summary of inconsistency estimates from different parts of the treatment network.	***19***
Risk of bias across studies	22	Present results of any assessment of risk of bias across studies for the evidence base being studied.	19
Results of additional analyses	23	Give results of additional analyses, if done (e.g., sensitivity or subgroup analyses, meta-regression analyses, alternative network geometries studied, alternative choice of prior distributions for Bayesian analyses, and so forth).	19

DISCUSSION

Summary of evidence	24	Summarize the main findings, including the strength of evidence for each main outcome; consider their relevance to key groups (e.g., healthcare providers, users, and policy-makers).	19-20
Limitations	25	Discuss limitations at study and outcome level (e.g., risk of bias), and at review level (e.g., incomplete retrieval of identified research, reporting bias). *Comment on the validity of the assumptions, such as transitivity and consistency. Comment on any concerns regarding network geometry (e.g., avoidance of certain comparisons).*	19-20

Conclusions	26	Provide a general interpretation of the results in the context of other evidence, and implications for future research.	1
**FUNDING**			
Funding	27	Describe sources of funding for the systematic review and other support (e.g., supply of data); role of funders for the systematic review. This should also include information regarding whether funding has been received from manufacturers of treatments in the network and/or whether some of the authors are content experts with professional conflicts of interest that could affect use of treatments in the network.	NA

**Table S2.Literature Search Strategy**	
**Pubmed**	((((((((((((((((((((Advanced Hepatocellular Carcinoma) OR Carcinomas, Hepatocellular) OR Hepatocellular Carcinomas) OR Hepatocellular Carcinoma) OR Hepatoma) OR Hepatomas) OR Liver Cancer, Adult) OR Adult Liver Cancer) OR Adult Liver Cancer) OR Cancer, Adult Liver) OR Cancers, Adult Liver) OR Liver Cancers, Adult) OR Liver Cell Carcinoma) OR Carcinoma, Liver Cell) OR Carcinomas, Liver Cell) OR Cell Carcinoma, Liver) OR Cell Carcinomas, Liver) OR Liver Cell Carcinomas) OR Liver Cell Carcinoma, Adult)) AND (((Nivolumab) OR Sorafenib) OR atezolizumab) OR Bevacizumab) OR Sintilimab) OR Pembrolizumab) OR Durvalumab) OR Tremelimumab) OR nivolumab) OR cabozantinib) OR donafenib) OR SIRT) OR cTACE) OR HAIC) OR pravastatin) OR GEMOX) OR Doxorubicin) OR erlotinib) OR Resminostat) OR Nintedanib) OR Lenvatinib) OR Tegafur–Uracil) OR HAI) OR Dovitinib) OR erlotinib) OR tigatuzumab) OR linifanib) OR sunitinib) OR Tslelizumab) OR camrelizumab–rivoceranib) OR PD-1)) OR drug targeting) OR Delivery System, Drug) OR Delivery Systems, Drug) OR Drug Delivery System) OR System, Drug Delivery) OR Systems, Drug Delivery) OR Drug Targeting) OR Drug Targetings) OR Targeting, Drug) OR Targetings, Drug) OR Checkpoint Inhibitors, Immune) OR Immune Checkpoint Blockers) OR Checkpoint Blockers, Immune) OR Immune Checkpoint Inhibitor) OR Checkpoint Inhibitor, Immune) OR CTLA-4 Inhibitors) OR CTLA 4 Inhibitors) OR Cytotoxic T-Lymphocyte-Associated Protein 4 Inhibitors) OR Cytotoxic T Lymphocyte Associated Protein 4 Inhibitors) OR Cytotoxic T-Lymphocyte-Associated Protein 4 Inhibitor) OR Cytotoxic T Lymphocyte Associated Protein 4 Inhibitor) OR CTLA-4 Inhibitor) OR CTLA 4 Inhibitor) OR PD-1 Inhibitors) OR PD 1 Inhibitors) OR Programmed Cell Death Protein 1 Inhibitor) OR Programmed Cell Death Protein 1 Inhibitors) OR PD-1 Inhibitor) OR Inhibitor, PD-1) OR PD 1 Inhibitor) OR Immune Checkpoint Blockade) OR Checkpoint Blockade, Immune) OR Immune Checkpoint Inhibition) OR Checkpoint Inhibition, Immune) OR PD-L1 Inhibitors) OR PD L1 Inhibitors) OR Programmed Death-Ligand 1 Inhibitors) OR Programmed Death Ligand 1 Inhibitors) OR PD-L1 Inhibitor) OR PD L1 Inhibitor) OR PD-1-PD-L1 Blockade)

	OR Blockade, PD-1-PD-L1) OR PD 1 PD L1 Blockade))) AND ((randomized controlled trail) OR randomized)
**Web of Science**	TS=(("hepatocellular carcinoma" OR hepatoma OR hcc OR "liver cancer" OR "liver cell carcinoma" OR "advanced hepatocellular carcinoma")<br>AND<br>("nivolumab" OR "sorafenib" OR "atezolizumab" OR "bevacizumab" OR "sintilimab" OR "pembrolizumab" OR "durvalumab" OR "tremelimumab" OR "cabozantinib" OR "donafenib" OR "pravastatin" OR "gemcitabine" OR "gemox" OR "doxorubicin" OR "erlotinib" OR "resminostat" OR<br>"nintedanib" OR "lenvatinib" OR "tegafur-uracil" OR "dovitinib" OR "tigatuzumab" OR "linifanib" OR "sunitinib" OR "tislelizumab" OR<br>"camrelizumab" OR "rivoceranib" OR "immune checkpoint inhibitor" OR "ctla-4 inhibitor" OR "pd-1 inhibitor" OR "pd-l1 inhibitor" OR<br>"immune checkpoint blockade" OR "drug targeting" OR "drug delivery system" OR "checkpoint inhibitor" OR "pd-1" OR "pd-l1" OR "ctla-4" OR<br>"immune checkpoint")<br>AND<br>(random* OR "randomized controlled trial" OR RCT))
**Cochrane**	(Advanced hepatocellular carcinoma OR Hepatocellular carcinoma OR Liver cancer OR Liver cell carcinoma OR Hepatoma)<br>AND<br>(Nivolumab OR Atezolizumab OR Bevacizumab OR Sintilimab OR Pembrolizumab OR Durvalumab OR Tremelimumab OR Cabozantinib OR Donafenib OR SIRT OR cTACE OR HAIC OR GEMOX OR Doxorubicin OR Erlotinib OR Resminostat OR Nintedanib OR Lenvatinib OR HAI OR Dovitinib OR Tigatuzumab OR Linifanib OR Sunitinib OR Tislelizumab OR Camrelizumab OR Rivoceranib OR PD-1 OR PD-L1 OR Immune Checkpoint Inhibitor OR CTLA-4)<br>AND<br>(Randomized OR "Randomized Controlled Trial")
**Embase**	('advanced hepatocellular carcinoma'/exp OR 'carcinoma, hepatocellular'/exp OR 'hepatoma'/exp OR 'liver cancer'/exp OR 'liver cell carcinoma'/exp OR hepatocellular carcinoma:ti,ab OR hepatoma:ti,ab OR hcc:ti,ab OR liver cancer:ti,ab)<br>AND<br>('nivolumab'/exp OR 'sorafenib'/exp OR 'atezolizumab'/exp OR 'bevacizumab'/exp OR 'sintilimab'/exp OR 'pembrolizumab'/exp OR 'durvalumab'/exp OR 'tremelimumab'/exp OR 'cabozantinib'/exp OR 'donafenib'/exp OR 'pravastatin'/exp OR 'gemcitabine'/exp OR 'doxorubicin'/exp OR 'erlotinib'/exp OR 'resminostat'/exp OR 'nintedanib'/exp OR 'lenvatinib'/exp OR 'tegafur uracil'/exp OR 'dovitinib'/exp OR 'tigatuzumab'/exp OR 'linifanib'/exp OR 'sunitinib'/exp OR 'tislelizumab'/exp OR 'camrelizumab'/exp OR 'rivoceranib'/exp OR 'immune checkpoint inhibitor'/exp OR 'ctla 4 inhibitor'/exp OR 'pd 1 inhibitor'/exp OR 'pd l1 inhibitor'/exp OR 'immune checkpoint blockade'/exp OR 'drug targeting'/exp OR 'drug delivery system'/exp OR 'checkpoint inhibitor'/exp OR nivolumab:ti,ab OR sorafenib:ti,ab OR atezolizumab:ti,ab OR bevacizumab:ti,ab OR sintilimab:ti,ab OR pembrolizumab:ti,ab OR durvalumab:ti,ab OR tremelimumab:ti,ab OR

	cabozantinib:ti,ab OR donafenib:ti,ab OR pravastatin:ti,ab OR gemox:ti,ab OR gemcitabine:ti,ab OR doxorubicin:ti,ab OR erlotinib:ti,ab OR resminostat:ti,ab OR nintedanib:ti,ab OR lenvatinib:ti,ab OR tegafur-uracil:ti,ab OR dovitinib:ti,ab OR tigatuzumab:ti,ab OR linifanib:ti,ab OR sunitinib:ti,ab OR tislelizumab:ti,ab OR camrelizumab:ti,ab OR rivoceranib:ti,ab OR 'pd-1':ti,ab OR 'pd-l1':ti,ab OR 'ctla-4':ti,ab OR 'immune checkpoint':ti,ab)<br>AND<br>(randomized controlled trial/exp OR random*:ti,ab OR rct:ti,ab)

Table S3. Consistency and Inconsistency Model Fit Comparison and Heterogeneity Assessment Across Endpoints in the Bayesian Network Meta-Analysis

Endpoints	Model Type	DIC	I^2
OS	Consistency	50.42	1%
	Inconsistency	51.29	3%
PFS	Consistency	28.08	7%
	Inconsistency	28.01	7%
ORR	Consistency	18.02	8%
	Inconsistency	18.01	8%
AE≥3	Consistency	15.50	9%
	Inconsistency	15.48	9%
HBV OS	Consistency	32.36	2%
	Inconsistency	32.99	3%
HCV OS	Consistency	28.07	7%
	Inconsistency	27.94	7%
NBNC OS	Consistency	17.90	11%
	Inconsistency	17.96	11%
HBV PFS	Consistency	18.07	11%
	Inconsistency	17.98	11%
HCV PFS	Consistency	16.00	12%
	Inconsistency	15.93	12%
NBNC PFS	Consistency	10.10	21%
	Inconsistency	10.01	20%

Table S4. Fixed-Effect vs Random-Effects Model Fit Comparison and Heterogeneity Assessment Across Endpoints in the Bayesian Network Meta-Analysis

Endpoints	Model Type	DIC	I^2
OS	Fixed-effect model	50.42	1.00%
	Random-effects model	51.11	1.00%
PFS	Fixed-effect model	28.08	7.00%
	Random-effects model	28.62	7.00%
ORR	Fixed-effect model	18.02	8.00%
	Random-effects model	18.62	8.00%
AE≥3	Fixed-effect model	15.50	9.00%
	Random-effects model	16.12	8.00%
HBV OS	Fixed-effect model	32.36	2.00%
	Random-effects model	33.01	2.00%
HCV OS	Fixed-effect model	28.07	7.00%
	Random-effects model	28.62	7.00%
NBNC OS	Fixed-effect model	17.90	11.00%
	Random-effects model	17.82	11.00%
HBV PFS	Fixed-effect model	18.07	11.00%
	Random-effects model	18.12	10.00%
HCV PFS	Fixed-effect model	16.00	12.00%
	Random-effects model	15.90	13.00%
NBNC PFS	Fixed-effect model	10.10	21.00%
	Random-effects model	9.30	22.00%

Table S5. Tumor Response Assessment Criteria and Baseline Liver Reserve Function (Across Trials)

Trial	ORR assessment criteria	Liver reserve function	Exp	Clt
IMbrave150	RECIST v1.1 (also mRECIST reported);Independent review facility	Child-Pugh class A only		
		Child-Pugh class score		
CheckMate 459	RECIST v1.1; Masked independent central review	5	227 (61%)	220 (59%)
		6	144 (39%)	152 (41%)
CARES-310	BIRC; RECIST v1.1 (mRECIST also described)	Child-Pugh class score		
		5	234 (86%)	226 (83%)
		6	38 (14%)	45 (17%)
		ALBI grade 1	127 (47%)	128 (47%)
		ALBI grade 2	145 (53%)	143 (53%)
RATIONALE-301	BIRC; RECIST v1.1	Child-Pugh class score		
		5	267 (78%)	267 (80%)
		6	75 (22%)	65 (20%)
		ALBI grade 1	187 (55%)	171 (52%)
		ALBI grade 2	155 (45%)	161 (48%)
COSMIC-312	BIRC; RECIST v1.1	Child-Pugh class score		
		ALBI grade 1	190 (44%)	102 (47%)
		ALBI grade 2	242 (56%)	115 (53%)
		ALBI grade 3	0.00%	0 (NR if not reported in your excerpt)

ORIENT-32	RECIST v1.1 (also mRECIST reported by IRRC); IRRC (masked) & investigator	Child-Pugh class score		
		A(5-6)	365 (96.1%)	182 (95.3%)
		B(7)	15 (3.9%)	9 (4.7%)
HIMALAYA	RECIST v1.1; Investigator assessed	Child-Pugh class A only		
LEAP-002	RECIST v1.1 by blinded independent central review	Child-Pugh class A only		
ALTN-AK105-III-02	RECIST v1.1 by IRC	NR		
CALGB 80802	NR	Child-Pugh class A only		
Thomas & Garrett-Mayer 2018	RECIST v1.1 by IRC	Child-Pugh class score		
		A(5-6)	7700.00%	
		B(7)	1300.00%	
Tak & Ryoo 2018	RECIST v1.1; Independent reviewer	Child-Pugh class score	Sorafenib	Resminostat + Sorafenib
		5	57 (67.9%)	61 (73.5%)
		6	26 (31.0%)	21 (25.3%)
		7	1 (1.2%)	1 (1.2%)
SILIUS	modified RECIST (mRECIST); Investigator assessed	Child-Pugh class score	Sorafenib	Sorafenib + HAIC
		5	59 (57%)	61 (60%)
		6	34 (33%)	29 (28%)
		7	10 (10%)	12 (12%)
Palmer & Ma 2018	RECIST v1.0 by central independent review	Child-Pugh class score	Nintedanib (n=62)	Sorafenib (n=31)

		5	42 (67.7%)	23 (74.2%)
		6	19 (30.6%)	8 (25.8%)
		7	1 (1.6%)	0 (0%)
REFLECT	RECIST v1.1	Child-Pugh class score	Lenvatinib	Sorafenib
		A	475 (99.4%)	471 (98.9%)
		B	3 (0.6%)	5 (1.1%)
ESLC01	RECIST v1.1 by independent radiologists	Child-Pugh class score	Sorafenib + UFT	Sorafenib
		A	35 (97.2%)	37 (97.4%)
		B7	1 (2.8%)	1 (2.6%)
Cheng & Thongprasert 2016	RECIST v1.1; Investigator assessed	Child-Pugh class score	Dovitinib	Sorafenib
		A	82 (100%)	82 (99%)
		B	0 (0%)	1 (1%)
SEARCH	RECIST v1.0; Investigator assessed	Child-Pugh class A only		
Cainap & Qin 2014	RECIST v1.1	Child-Pugh class score	Linifanib	Sorafenib
		A	484 (93.2%)	493 (95.0%)
		B	30 (5.8%)	26 (5.0%)
Cheng & Kang 2015	RECIST v1.1; investigator assessed	Child-Pugh class A only		
BRISK-FL	mRECIST	Child-Pugh class score	Sorafenib (n=578)	Brivanib (n=577)
		A	531 (92%)	531 (92%)
		B	47 (8%)	46 (8%)
SUN 1170	RECIST v1.0 investigator assessed	Child-Pugh class score	Sunitinib (n=530)	Sorafenib (n=544)
		A (5-6)	529 (99.8%)	541 (99.4%)

		Missing	1 (0.2%)	3 (0.6%)
NRG/RTOG 1112	NR	Child-Pugh class A only		
Donafenib vs Sorafenib 2021	RECIST v1.1; Investigator assessed; Independent Review Committee (IRC)	Child-Pugh class score	Donafenib (n = 328)	Sorafenib (n = 331)
		5	262 (80%)	259 (78%)
		6	62 (19%)	59 (18%)
		7	4 (1%)	13 (4%)

Table S6. Etiology Subgroups (Experimental vs Control)

Trial	Etiology subgroups (Exp vs Ctrl)	Exp	Clt
IMbrave150	Etiology	Atezolizumab+Bevacizumab	Sorafenib
	HBV	164 (48.8%)	76 (46.1%)
	HCV	72 (21.4%)	36 (21.8%)
	Non-viral	100 (29.8%)	53 (32.1%)
CheckMate 459	Etiology	Nivolumab	Sorafenib
	HBV	177 (48%)	158 (42%)
	HCV	77 (21%)	90 (24%)
	Non-viral	117 (32%)	124 (33%)
CARES-310	Etiology	Camrelizumab+Rivoceranib	Sorafenib
	HBV	188 (69%)	180 (66%)
	HCV	35 (13%)	42 (15%)
	Uninfected	49 (18%)	49 (18%)
RATIONALE-301	Etiology	Tislelizumab	Sorafenib
	HBV	140 (41%)	132 (40%)

	HCV	38 (11%)	44 (13%)
	HBV+HCV	3 (1%)	1 (<1%)
	Uninfected	149 (44%)	155 (47%)
COSMIC-312	Etiology	Cabozantinib+ Atezolizumab	Sorafenib
	HBV	212 (49%)	100 (46%)
	HCV	114 (26%)	55 (25%)
	Non-viral	106 (25%)	62 (29%)
ORIENT-32	Etiology	Sintilimab+IBI305	Sorafenib
	HBV positive	359 (94.5%)	179 (93.7%)
	HBV negative	21 (5.5%)	12 (6.3%)
	(HCV breakdown NR)		
HIMALAYA	Etiology	STRIDE (n=103)/Durvalumab (n=79)	Sorafenib (n=64)
	HBV	33 (32.0%)/25 (31.6%)	16 (25.0%)
	HCV	30 (29.1%)/23 (29.1%)	26 (40.6%)
	Non-viral	40 (38.8%)/31 (39.2%)	22 (34.4%)
LEAP-002	NR		
ALTN-AK105-III-02	NR		
CALGB 80802	HCV-positive at baselin: 83 / 356		
Thomas & Garrett-Mayer 2018	NR		
Tak & Ryoo 2018	Etiology	Sorafenib	Resminostat + Sorafenib
	HBV	46 (54.8%)	44 (53.0%)
	HCV	23 (27.4%)	11 (13.3%)
SILIUS	Etiology	Sorafenib	Sorafenib + HAIC
	HBV	22 (21%)	26 (26%)
	HCV	46 (45%)	47 (46%)
	Non-viral / other	35 (34%)	29 (28%)

Palmer & Ma 2018	Etiology	Nintedanib	Sorafenib
	HBV-related	4 (6.5%)	7 (22.6%)
	HCV-related	13 (21.0%)	8 (25.8%)
REFLECT	Etiology	Lenvatinib	Sorafenib
	HBV	251 (52.5%)	228 (47.9%)
	HCV	91 (19.0%)	126 (26.5%)
ESLC01	Etiology	Sorafenib + UFT	Sorafenib
	HCV positive	31 (86.1%)	33 (86.8%)
	HBV positive	0 (0.0%)	1 (2.6%)
	HBV + HCV	0 (0.0%)	3 (7.9%)
	Non-viral	5 (13.9%)	1 (2.6%)
Cheng & Thongprasert 2016	Etiology	Dovitinib	Sorafenib
	HBV infection	72%	64%
SEARCH	Etiology	Sorafenib + Placebo	Sorafenib + Erlotinib
	HBV	133 (37.2%)	122 (33.7%)
	HCV	84 (23.5%)	107 (29.6%)
	Non-viral / other	141 (39.4%)	133 (36.7%)
Cainap & Qin 2014	Etiology	Linifanib	Sorafenib
	HBV	275 (53.5%)	276 (53.0%)
	HCV	130 (25.3%)	129 (24.8%)
Cheng & Kang 2015	Etiology	TIG 6/2 + SOR (n=53)/TIG 6/6 + SOR (n=54)	SOR (n=55)
	HBV	32 (60.4%)/24 (44.4%)	25 (45.5%)
	HCV	16 (30.2%)/20 (37.0%)	19 (34.5%)
BRISK-FL	Etiology	Sorafenib (n=578)	Brivanib (n=577)
	HBV	258 (45%)	254 (44%)
	HCV	119 (21%)	116 (20%)

	Etiology	Sunitinib (n=530)	Sorafenib (n=544)
SUN 1170	HBV infection	290 (54.7%)	288 (52.9%)
	HCV infection	113 (21.3%)	119 (21.9%)
NRG/RTOG 1112	Etiology		
	HCV	41%	
	Hepatitis B or B/C	19%	
	Etiology	Donafenib (n = 328)	Sorafenib (n = 331)
	HBV	293 (89%)	301 (91%)
Donafenib vs Sorafenib 2021	HCV	7 (2%)	5 (2%)
	NAFLD	0	2 (1%)
	Others	28 (9%)	23 (7%)

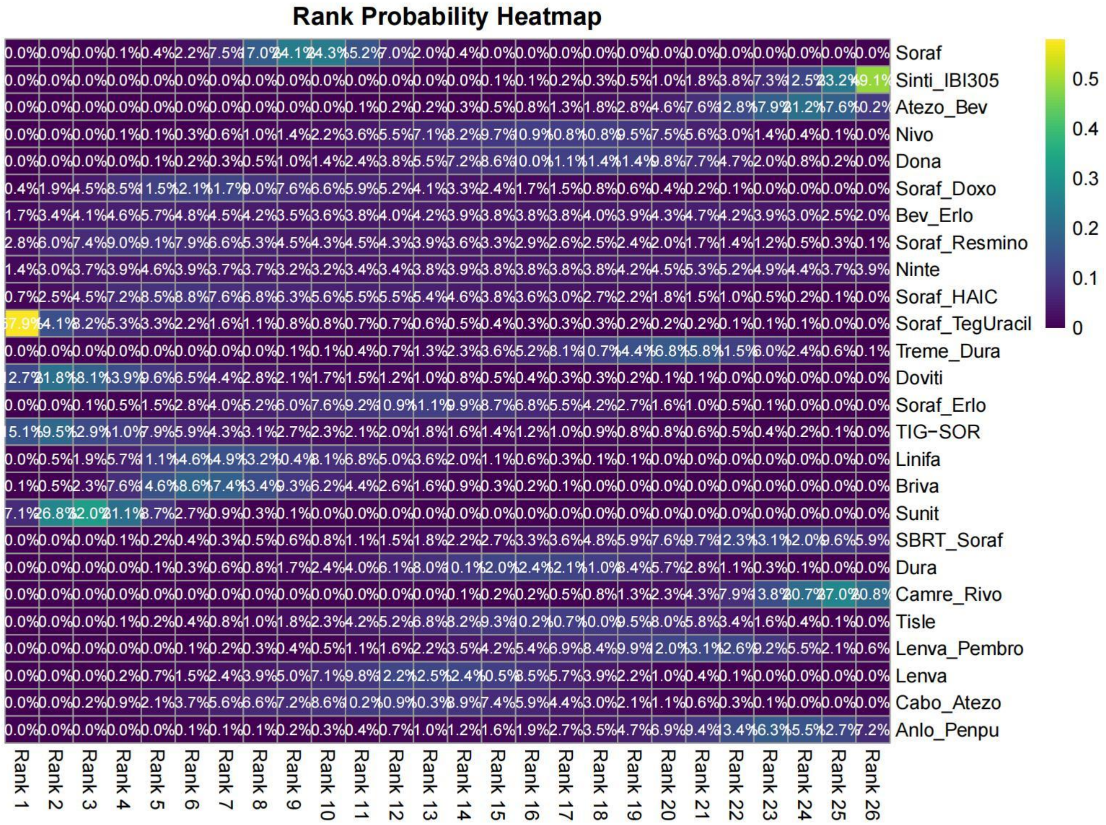

Figure S1.Rank Probability Heatmap of Treatment Regimens for OS in Advanced HCC.

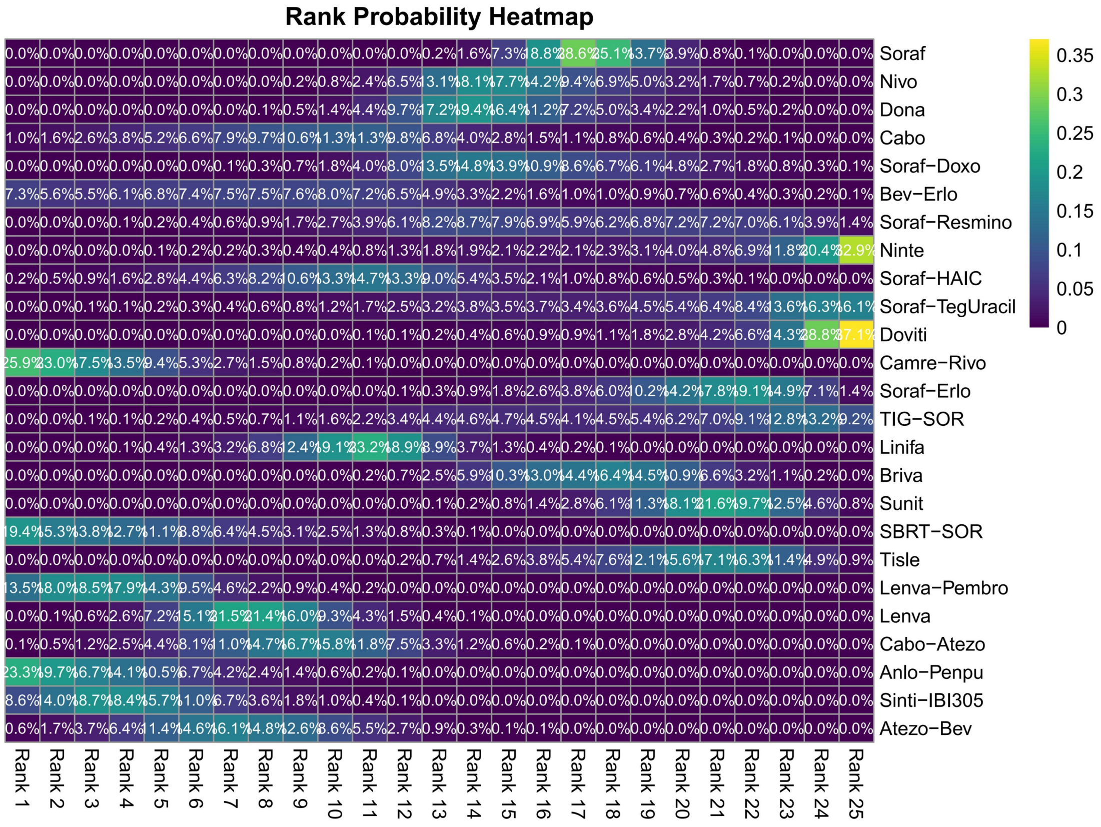

Figure S2.Rank Probability Heatmap of Treatment Regimens for PFS in Advanced HCC.

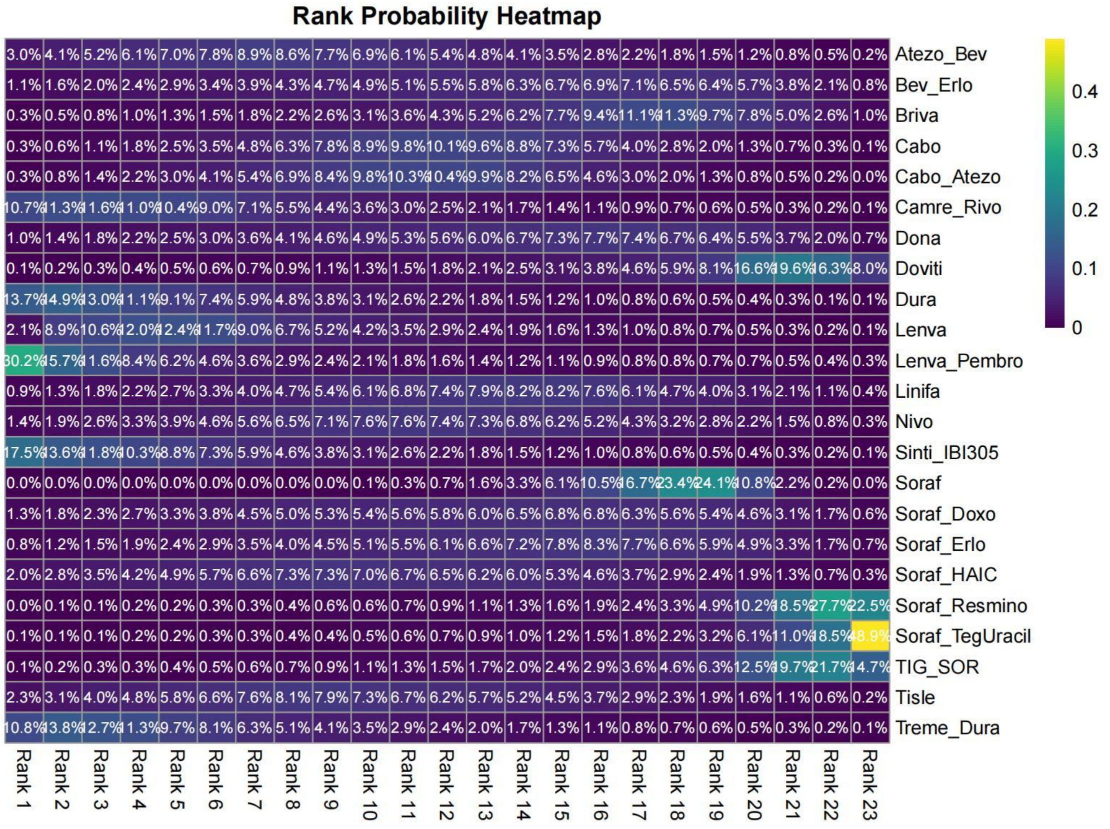

Figure S3.Rank Probability Heatmap of Treatment Regimens for ORR in Advanced HCC.

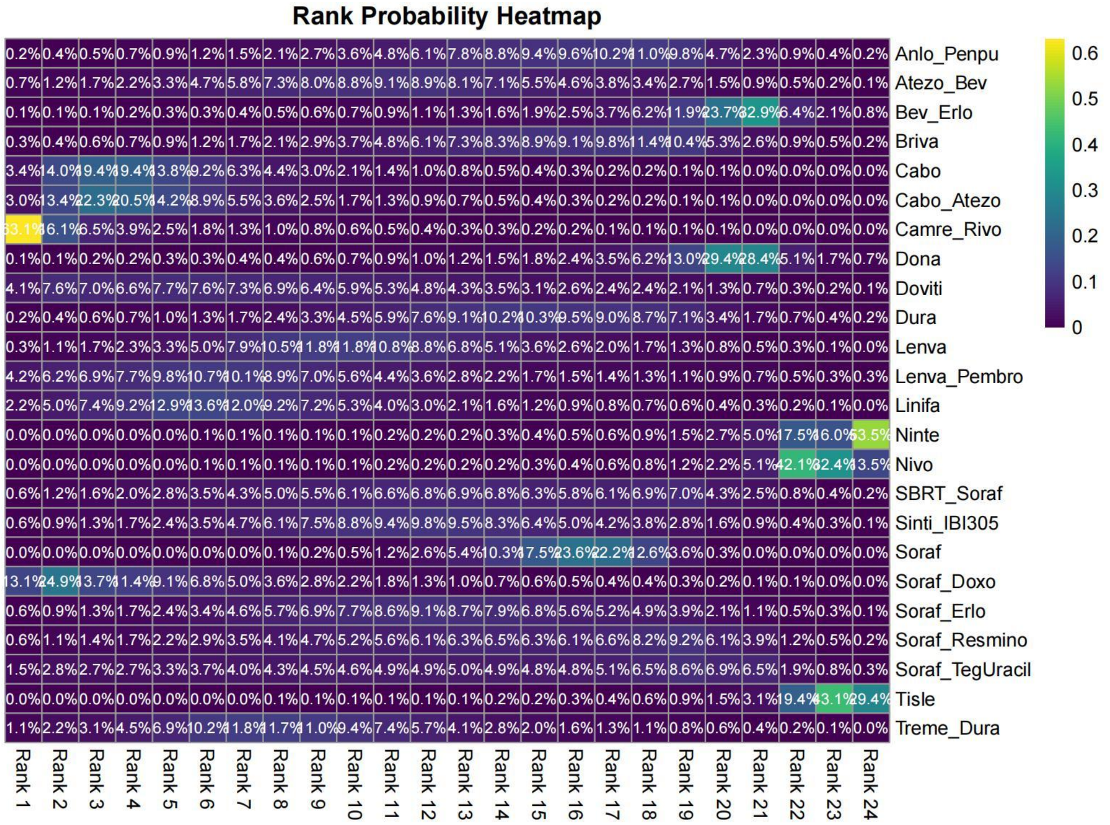

Figure S4.Rank Probability Heatmap of Treatment Regimens for AE≥3 in Advanced HCC.

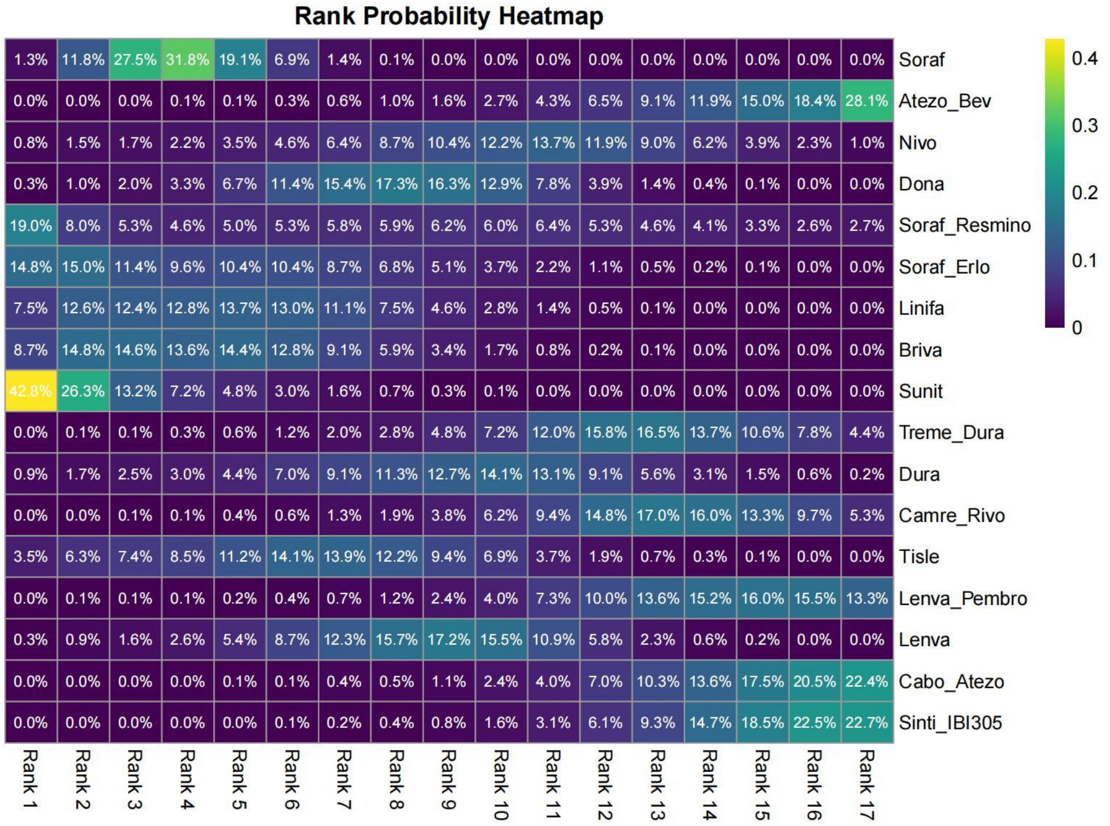

Figure S5.Rank Probability Heatmap of Treatment Regimens for OS in HBV-positive Advanced HCC.

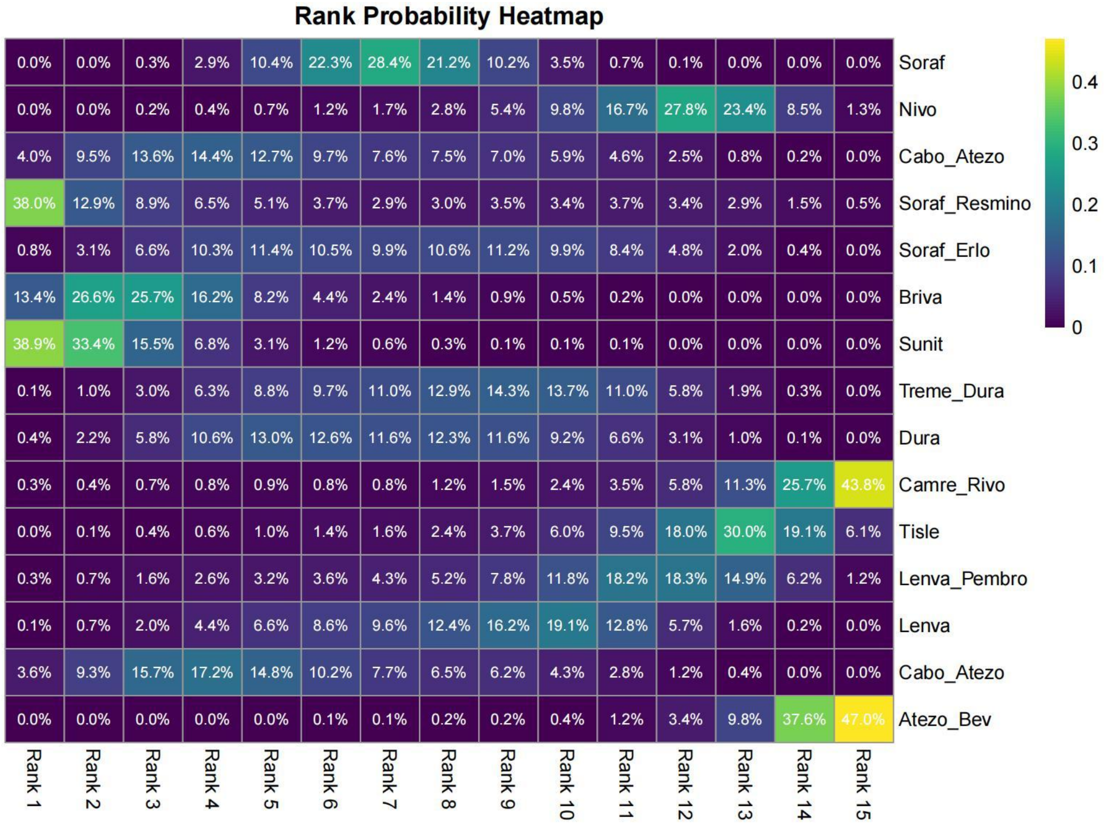

Figure S6.Rank Probability Heatmap of Treatment Regimens for OS in HCV-positive Advanced HCC.

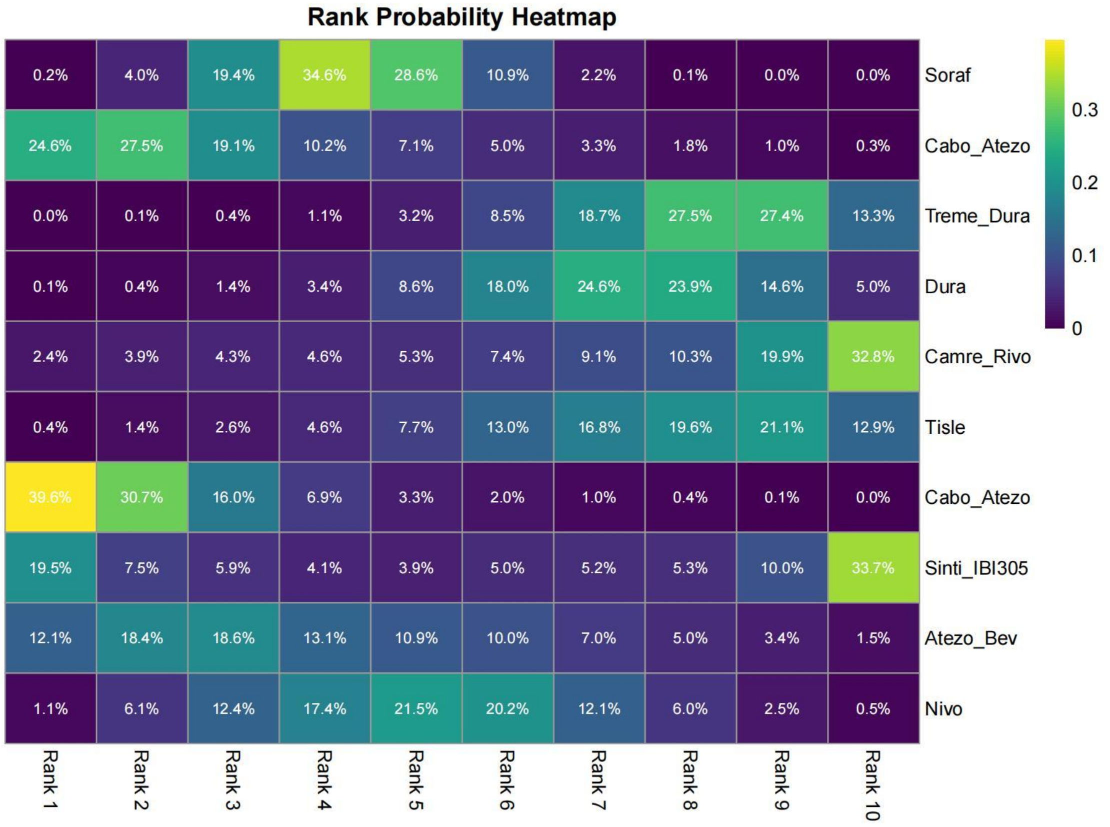

Figure S7.Rank Probability Heatmap of Treatment Regimens for OS in NBNCAdvanced HCC.

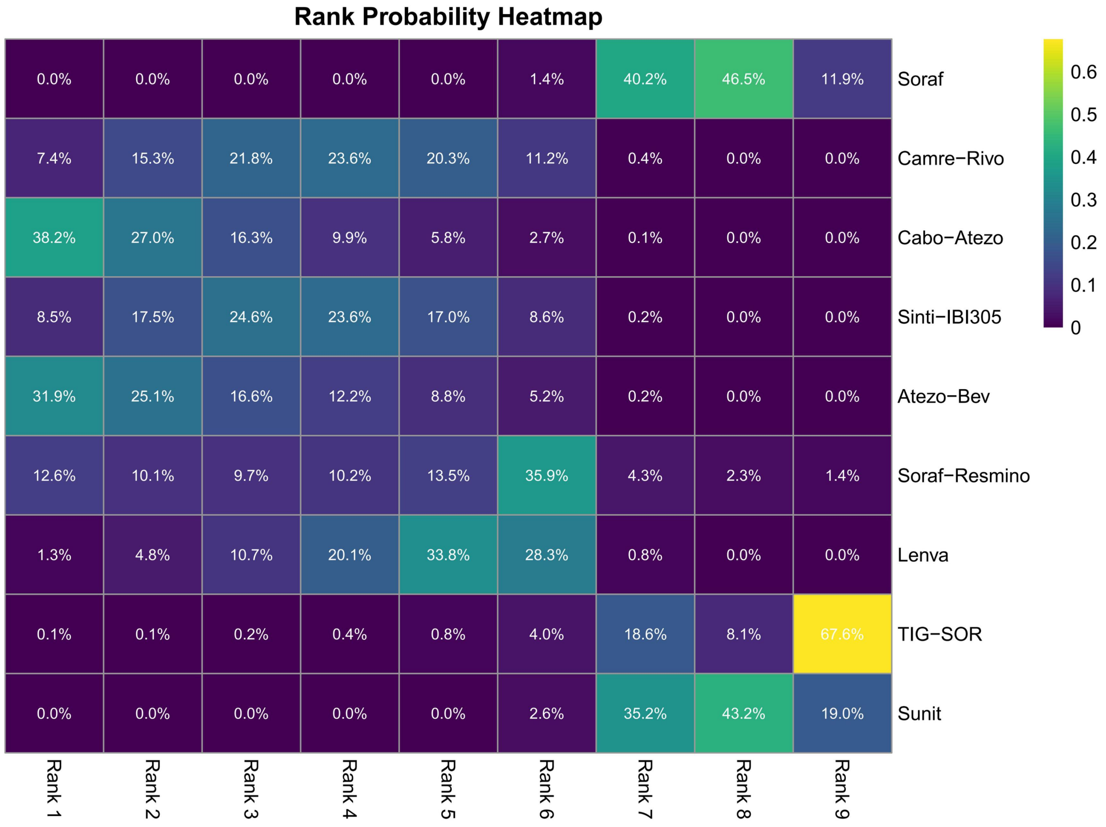

Figure S8.Rank Probability Heatmap of Treatment Regimens for PFS in HBV-positive Advanced HCC.

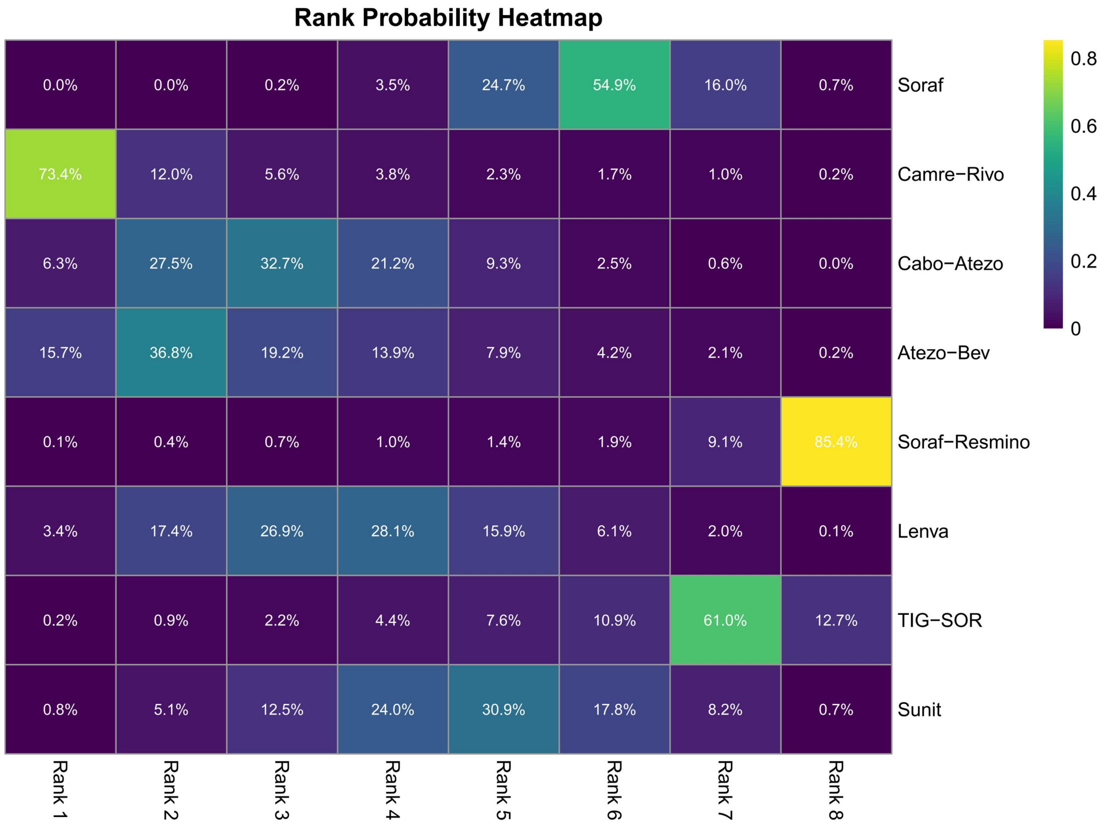

Figure S9.Rank Probability Heatmap of Treatment Regimens for PFS in HCV-positive Advanced HCC.

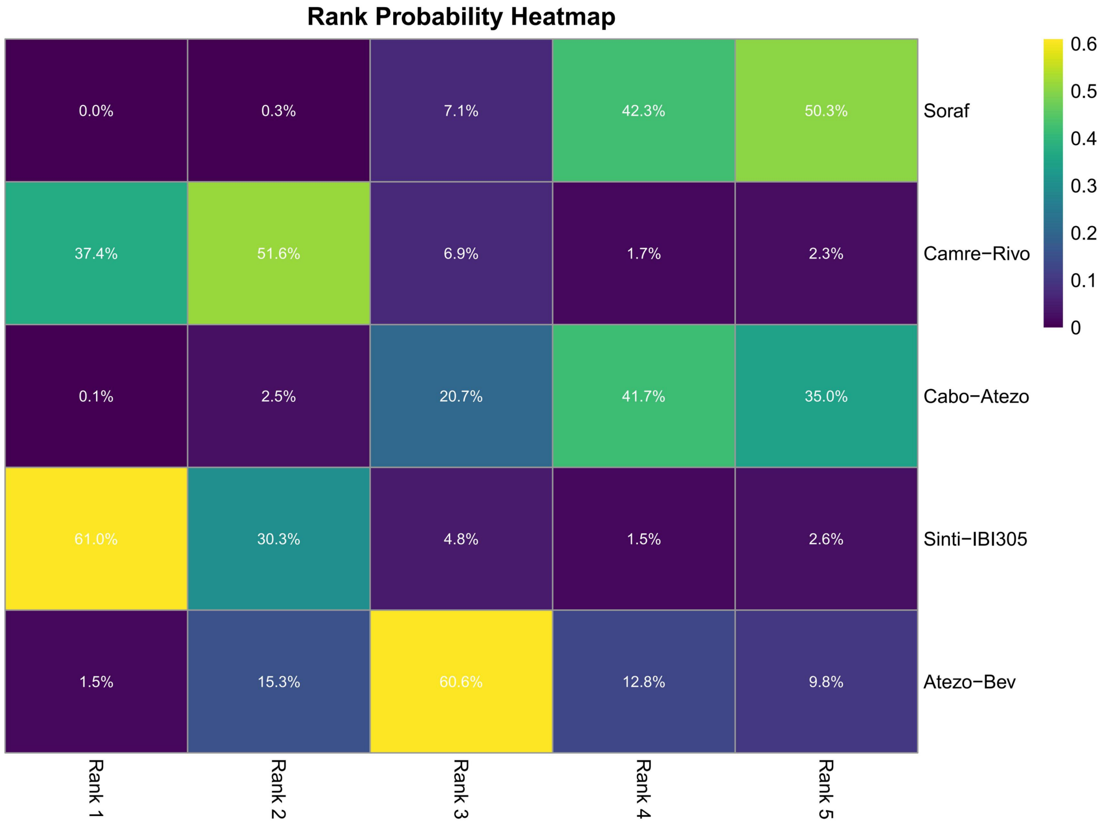

Figure S10.Rank Probability Heatmap of Treatment Regimens for PFS in NBNC Advanced HCC.

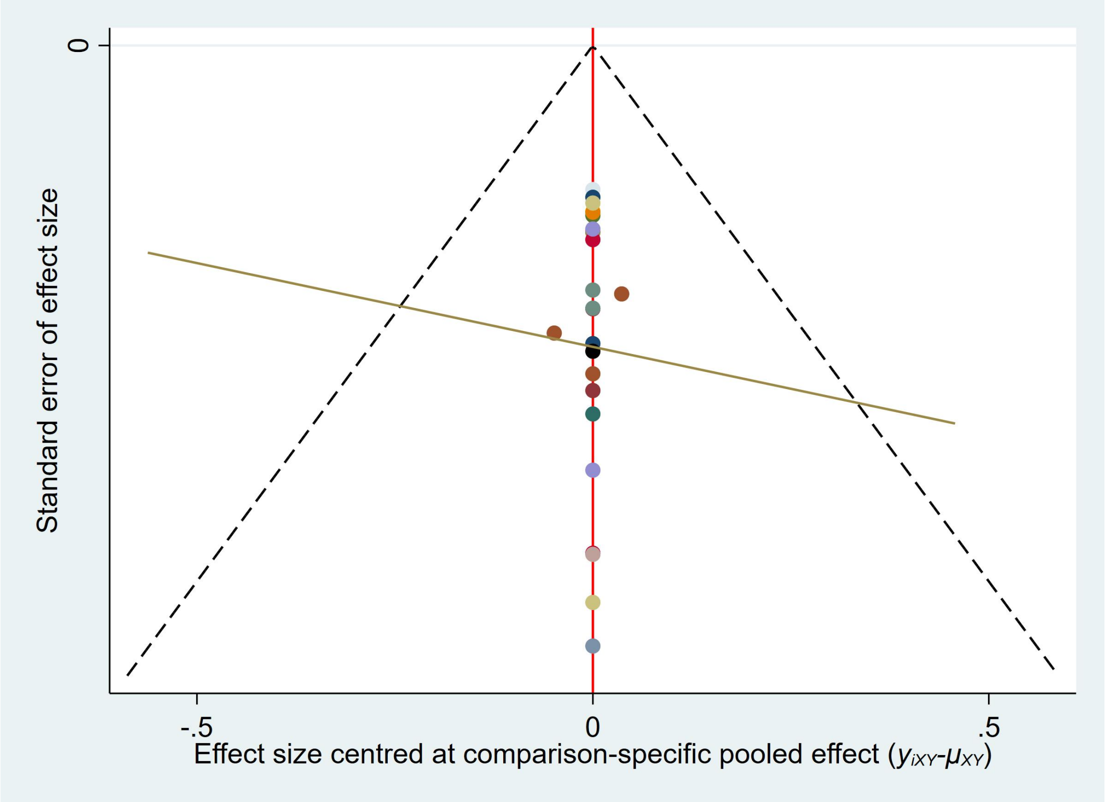

Figure S11.Comparison adjusted funnel plot for overall survival in first line therapie

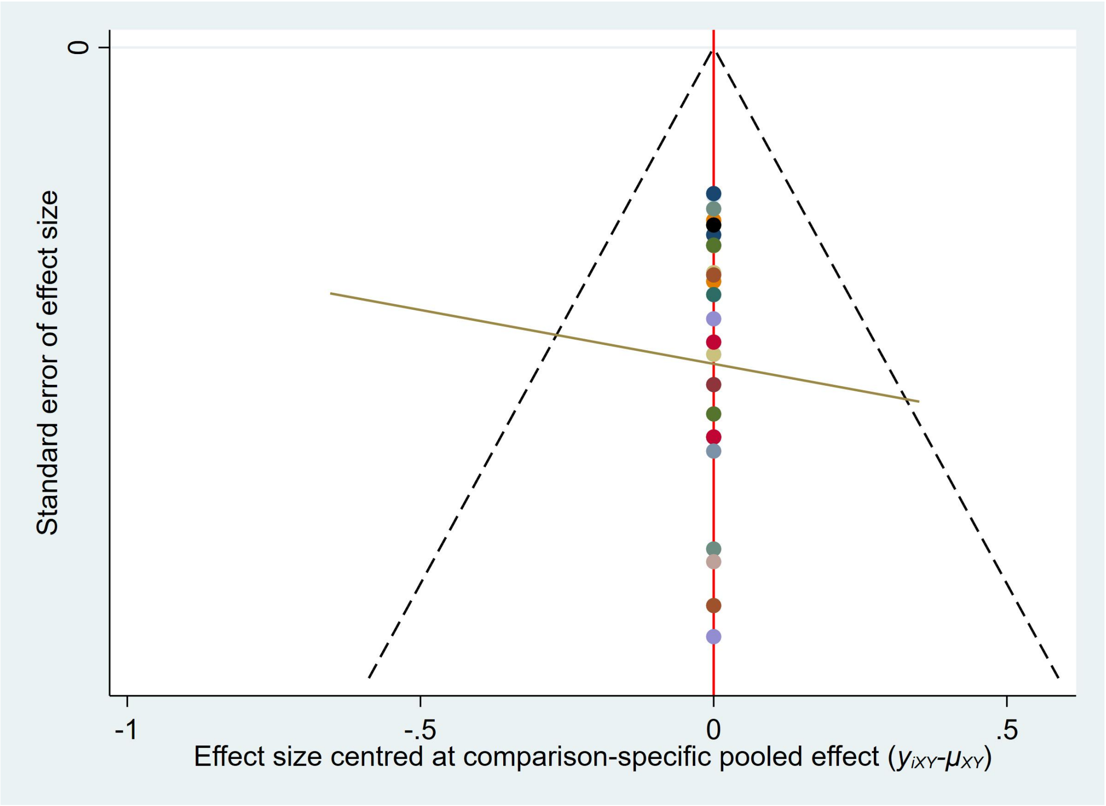

Figure S12.Comparison adjusted funnel plot for progression free survival in first line therapies

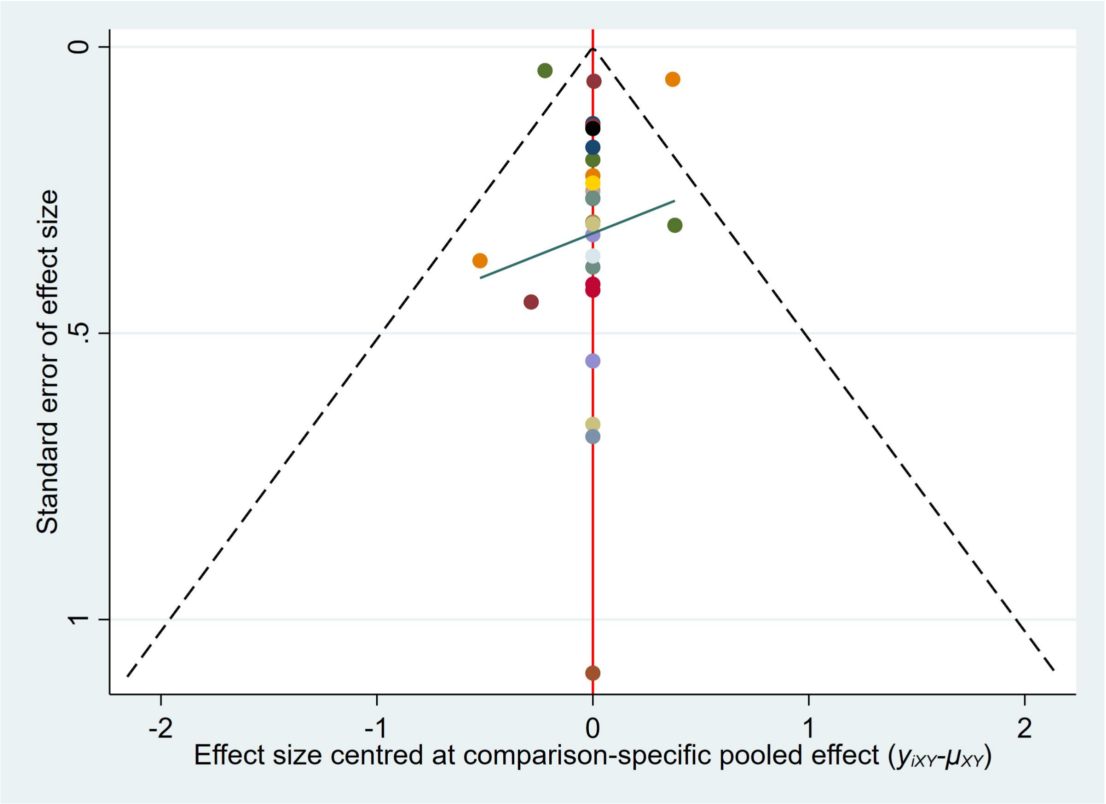

Figure S13.Comparison adjusted funnel plot for objective response rate in first line therapies

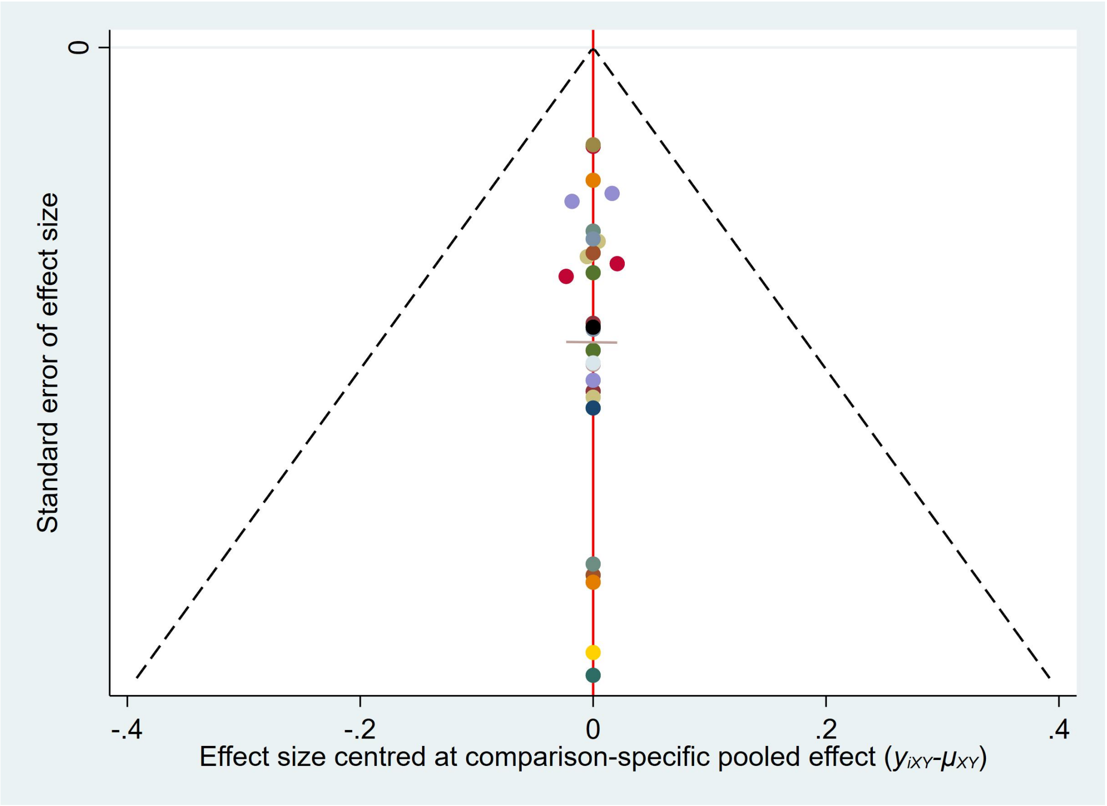

Figure S14.Comparison adjusted funnel plot for grade ≥3 adverse events in first line therapies

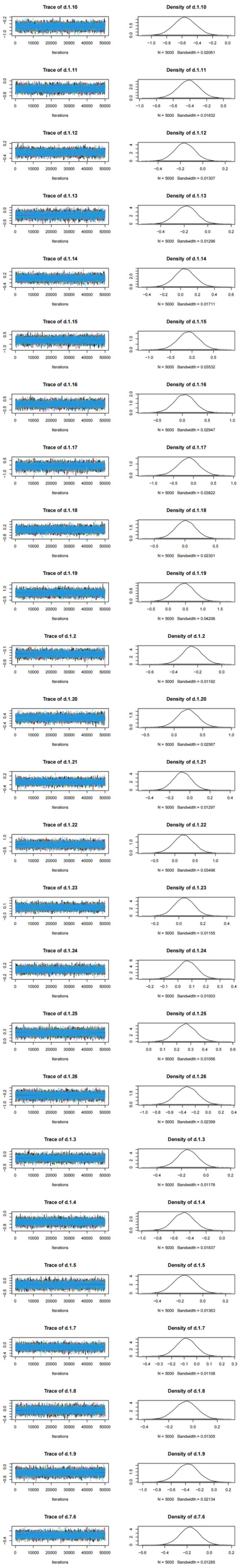

Figure S15.MCMC trace and posterior density plots for OS in advanced hepatocellular carcinoma

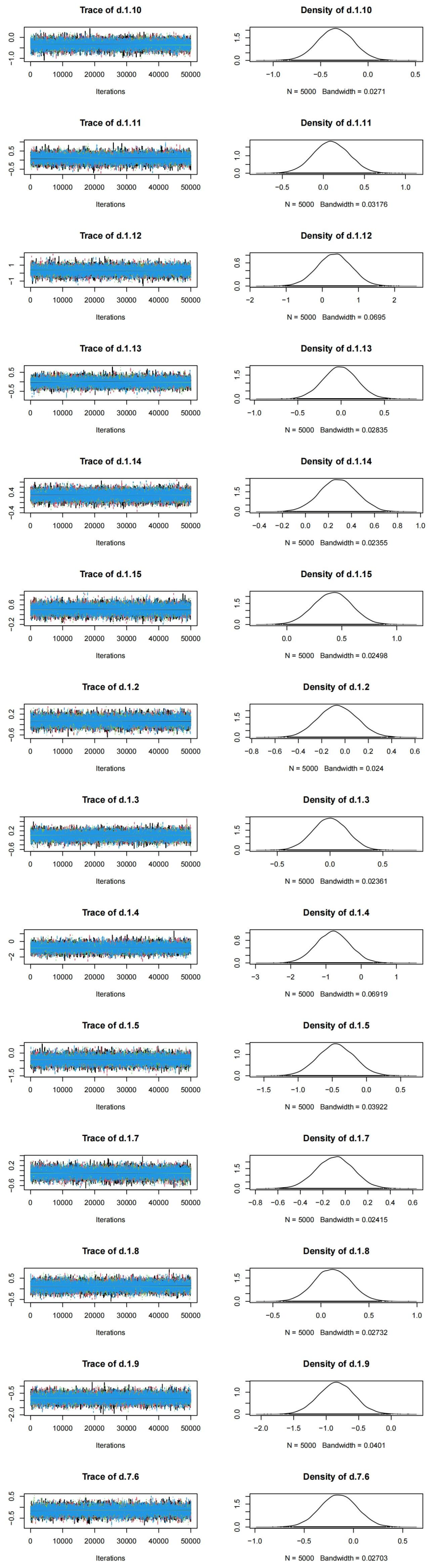

Figure S16.MCMC trace and posterior density plots for PFS in advanced hepatocellular carcinoma

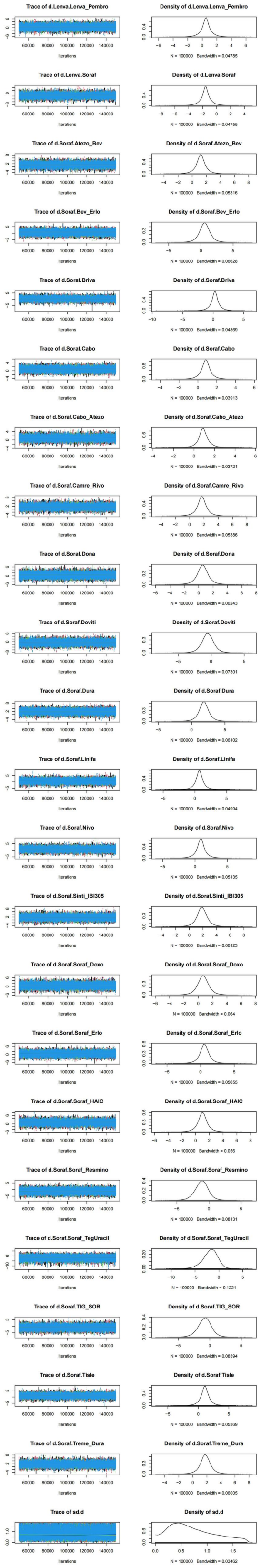

Figure S17.MCMC trace and posterior density plots for ORR in advanced hepatocellular carcinoma

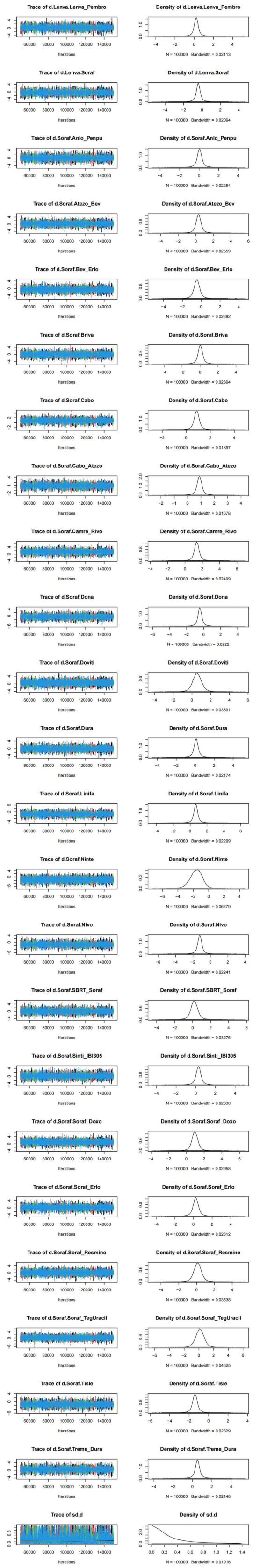

Figure S18.MCMC trace and posterior density plots for AEs ⩾ 3 in advanced hepatocellular carcinoma

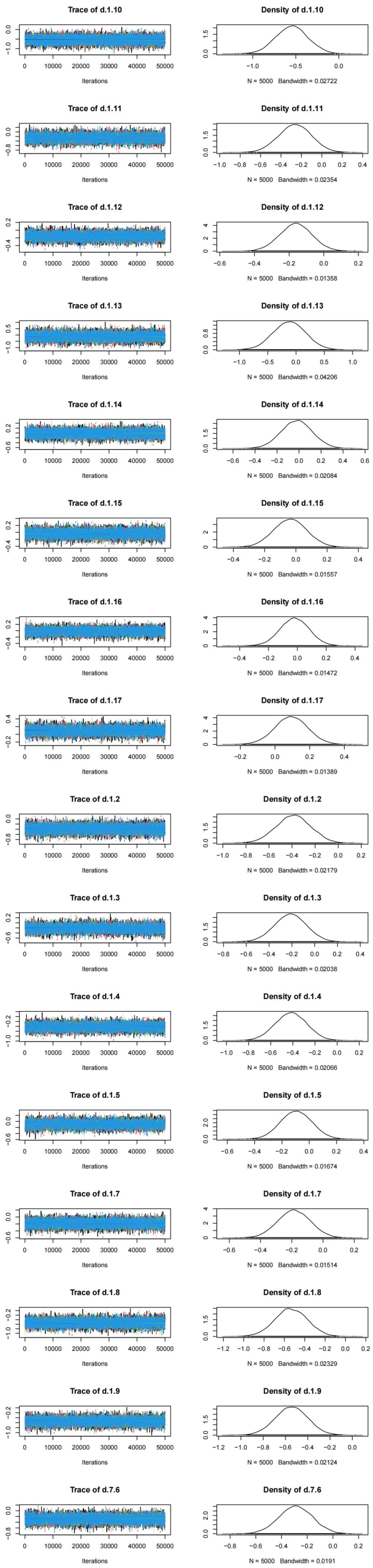

Figure S19.MCMC trace and posterior density plots for OS in HBV-positive advanced hepatocellular carcinoma

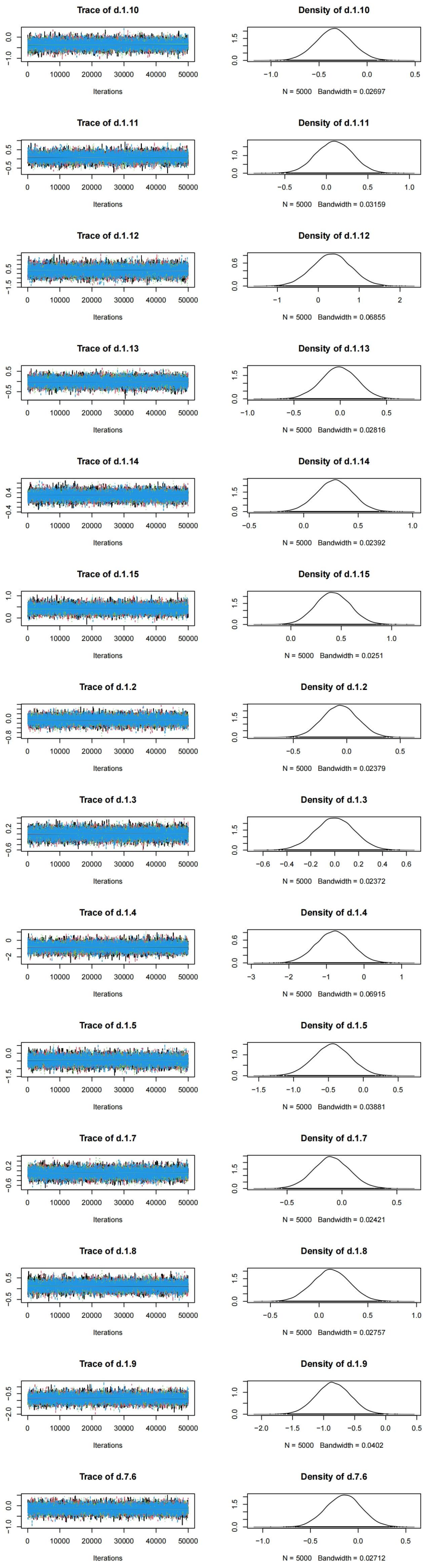

Figure S20.MCMC trace and posterior density plots for OS in HCV-positive advanced hepatocellular carcinoma

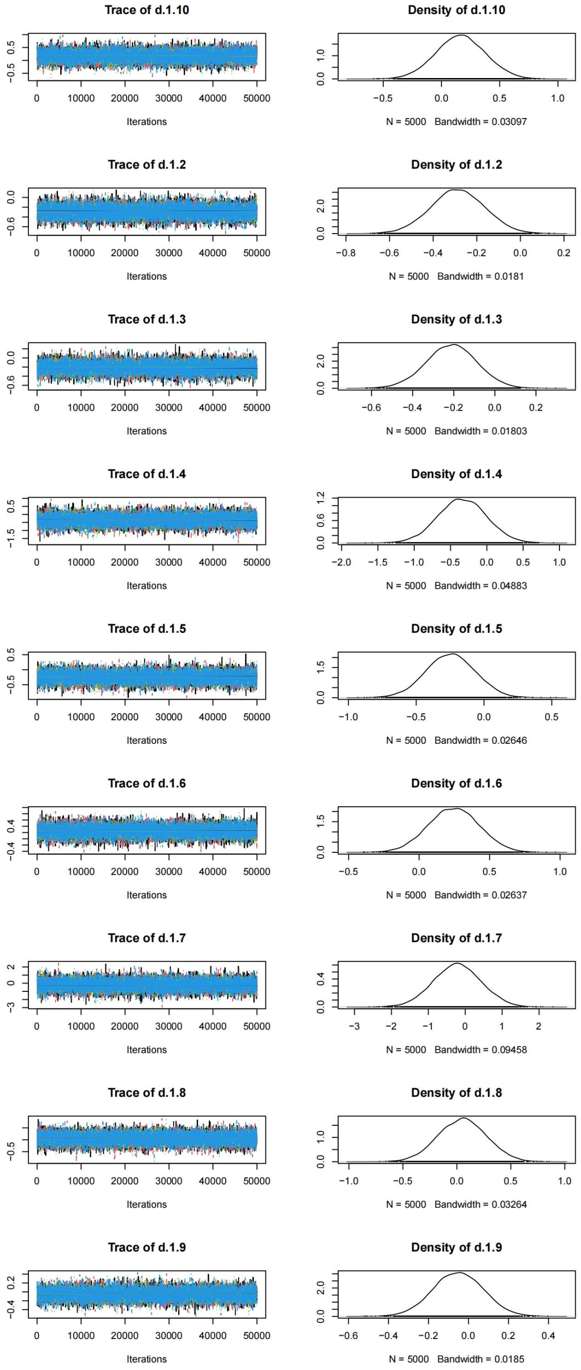

Figure S21.MCMC trace and posterior density plots for OS in NBNC advanced hepatocellular carcinoma

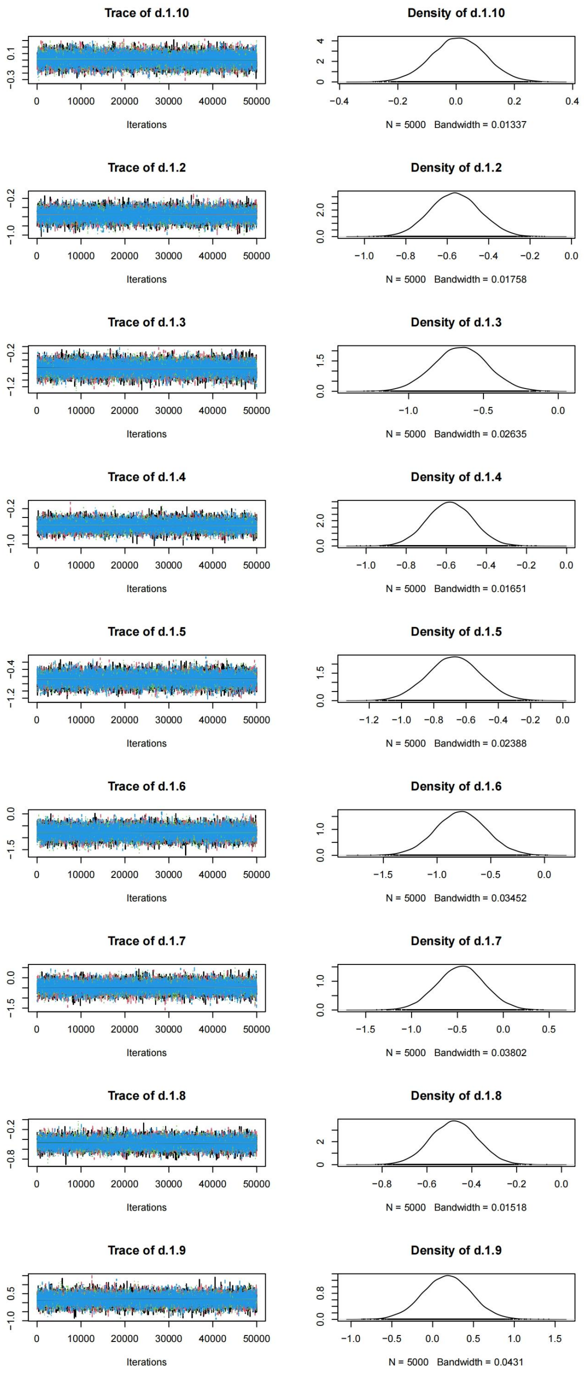

Figure S22.MCMC trace and posterior density plots for PFS in HBV-positive advanced hepatocellular carcinoma

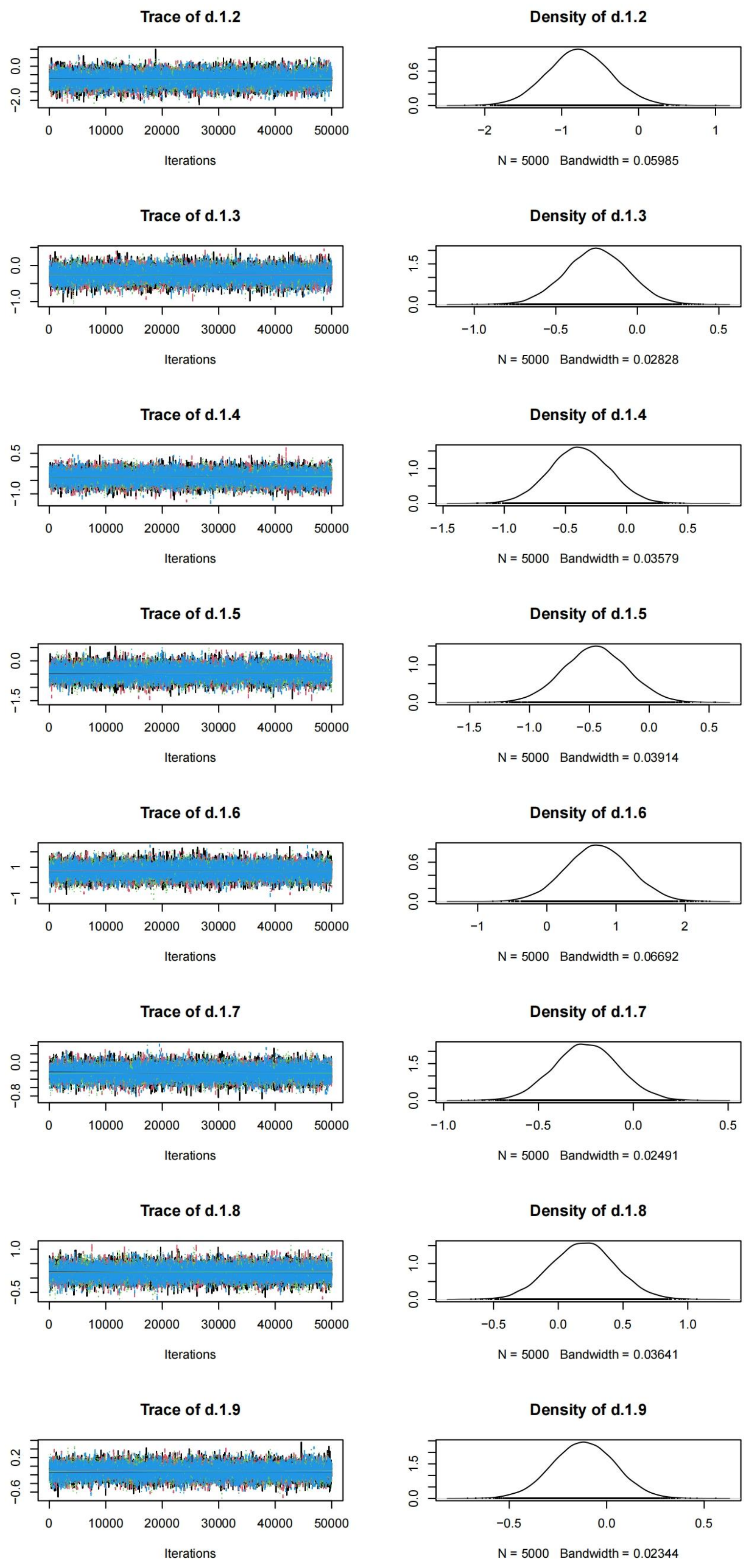

Figure S23.MCMC trace and posterior density plots for PFS in HCV-positive advanced hepatocellular carcinoma

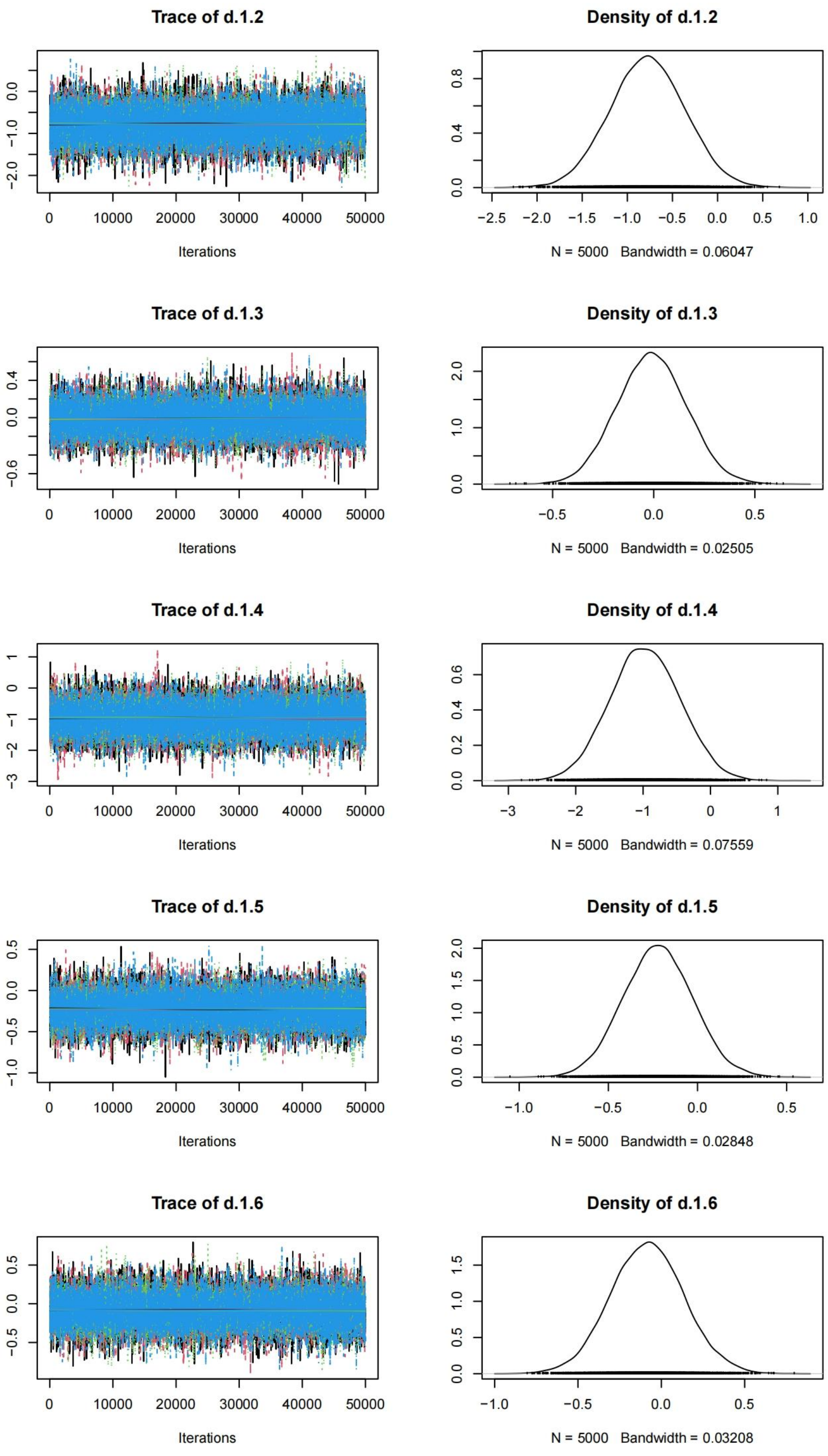

Figure S24.MCMC trace and posterior density plots for PFS in NBNC advanced hepatocellular carcinoma

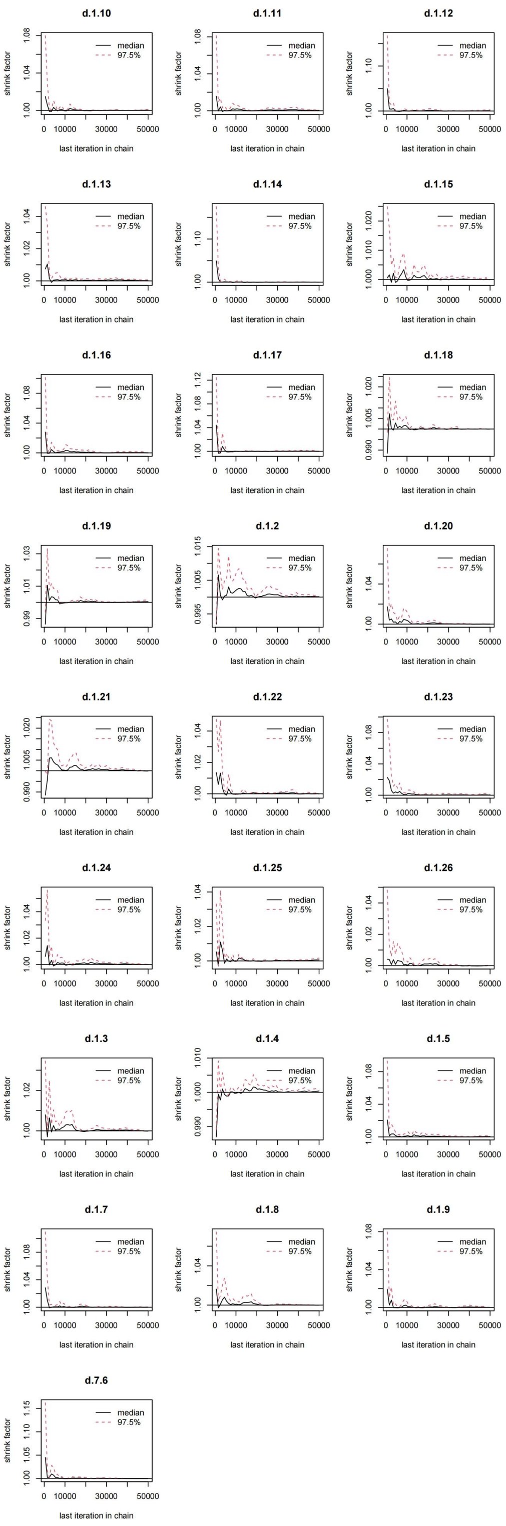

Figure S25.Convergence diagnostics for OS in advanced hepatocellular carcinoma

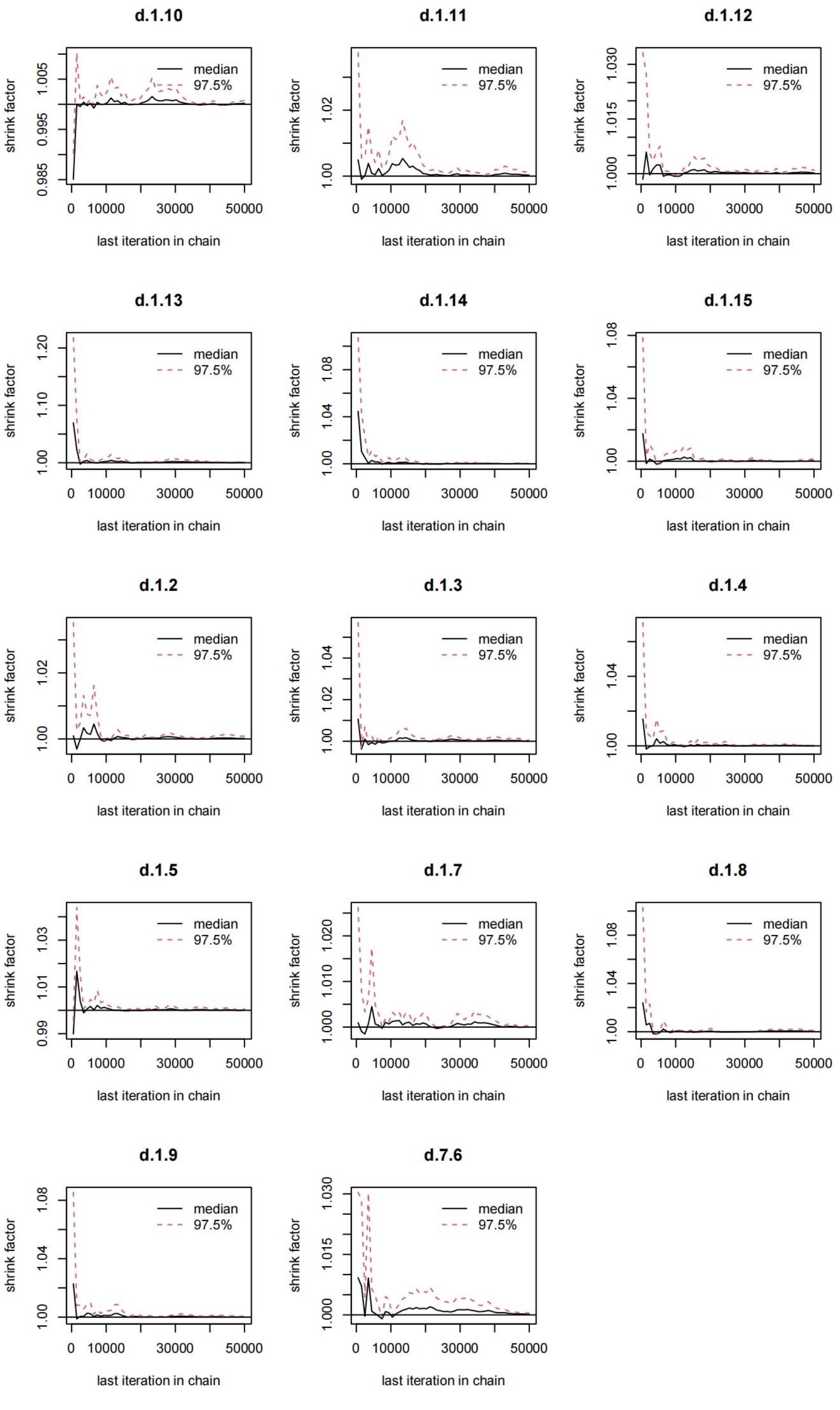

Figure S26.Convergence diagnostics for PFS in advanced hepatocellular carcinoma

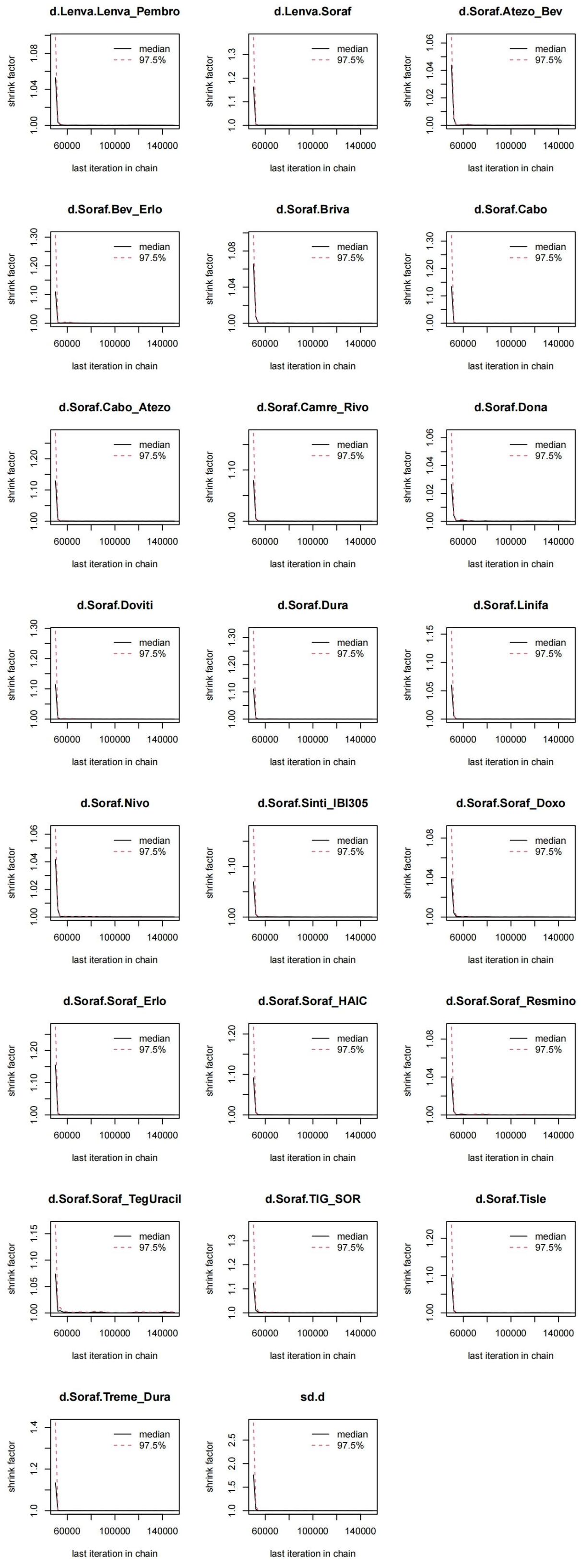

Figure S27.Convergence diagnostics for ORR in advanced hepatocellular carcinoma

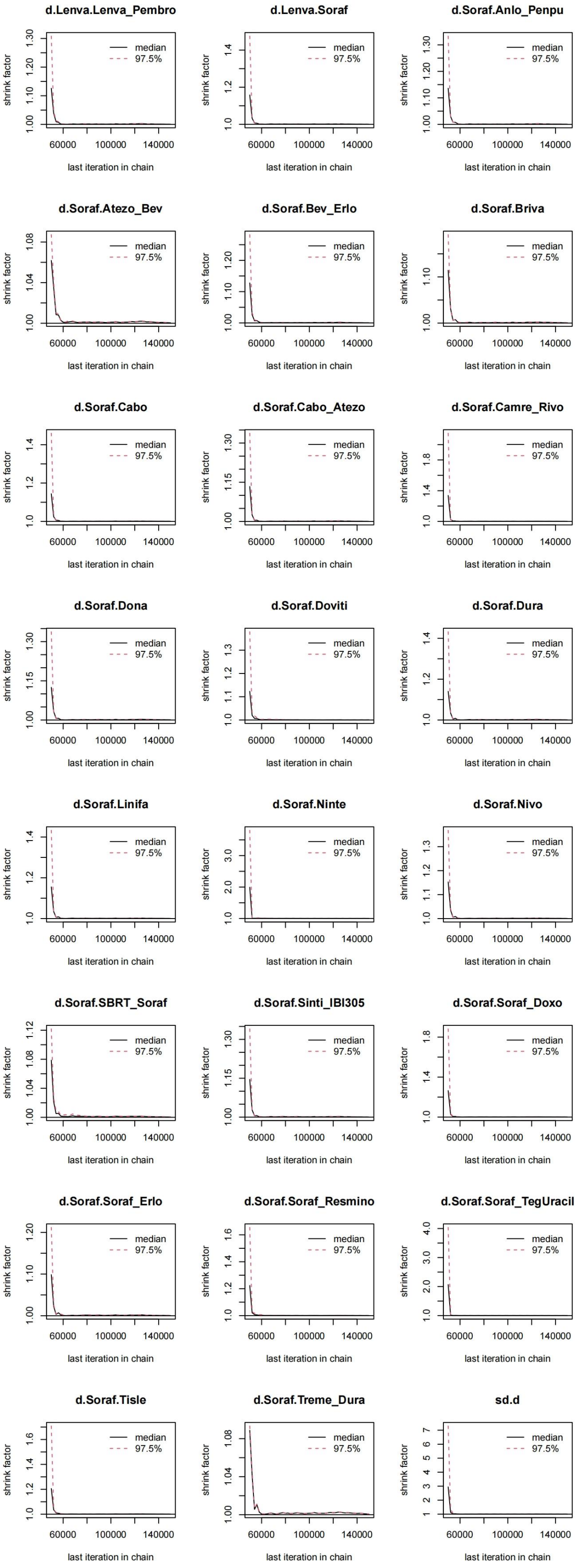

Figure S28.Convergence diagnostics for AEs≥3 in advanced hepatocellular carcinoma

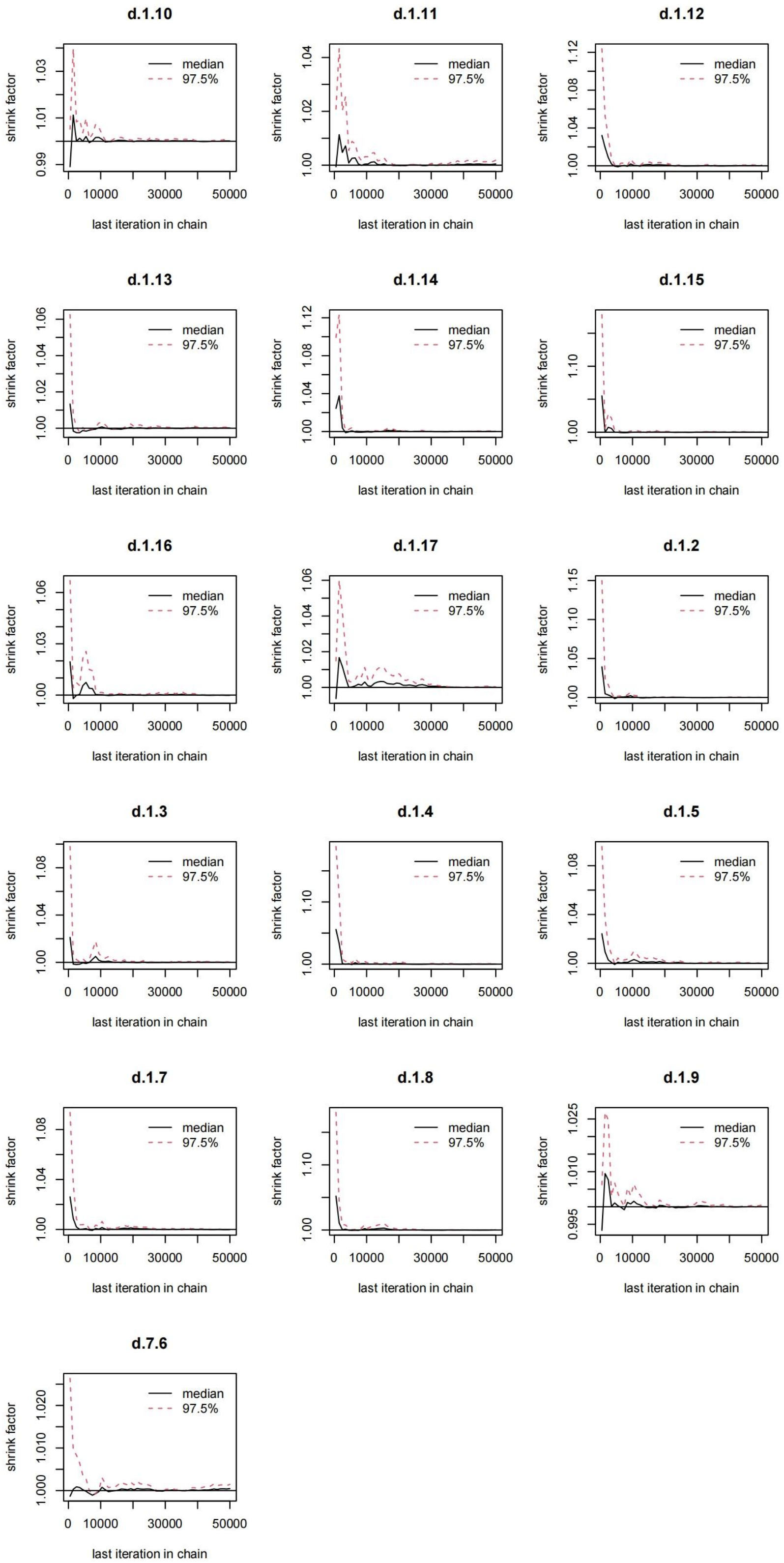

Figure S29.Convergence diagnostics for OS in HBV-positive advanced hepatocellular carcinoma

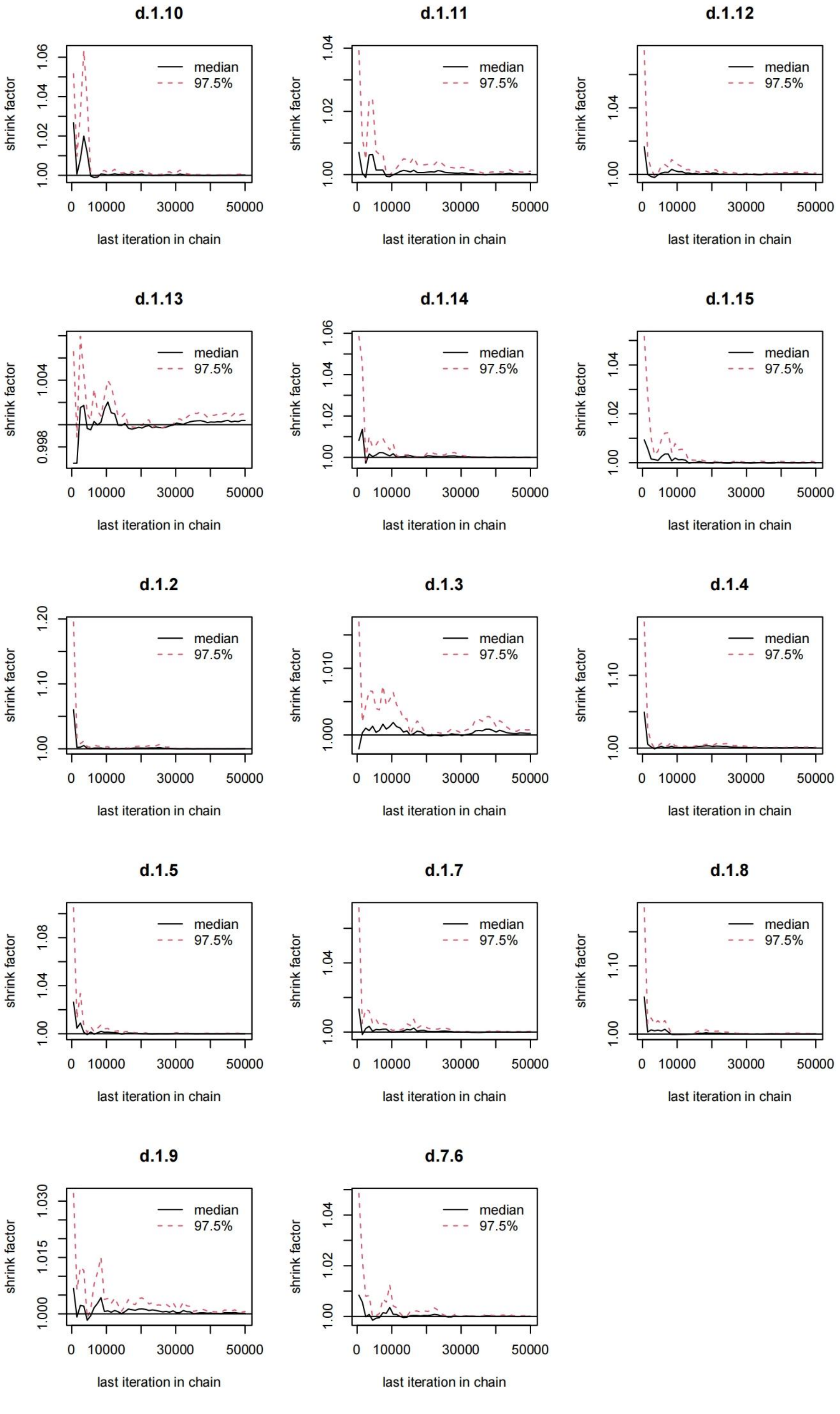

Figure S30.Convergence diagnostics for OS in HCV-positive advanced hepatocellular carcinoma

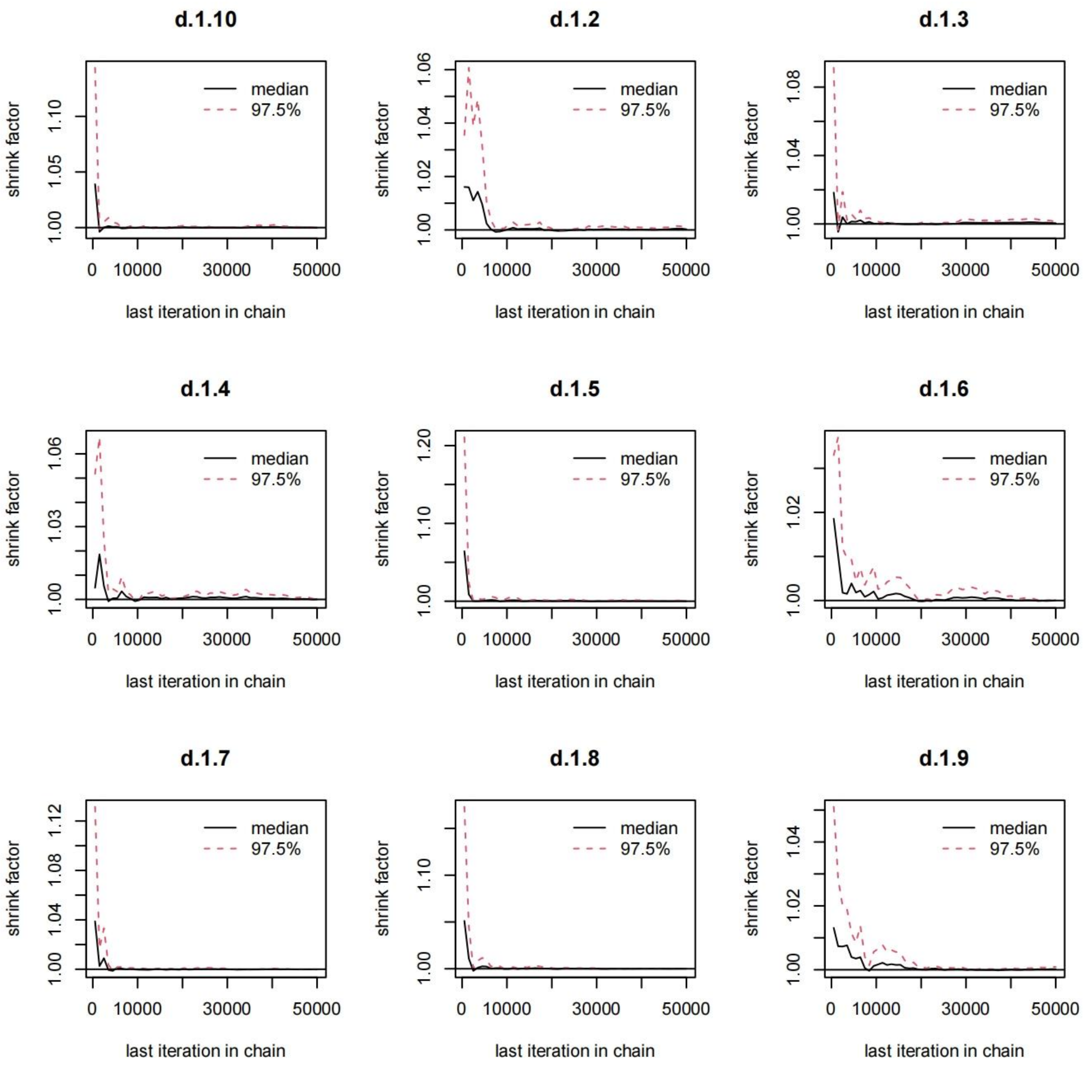

Figure S31.Convergence diagnostics for OS in NBNC advanced hepatocellular carcinoma

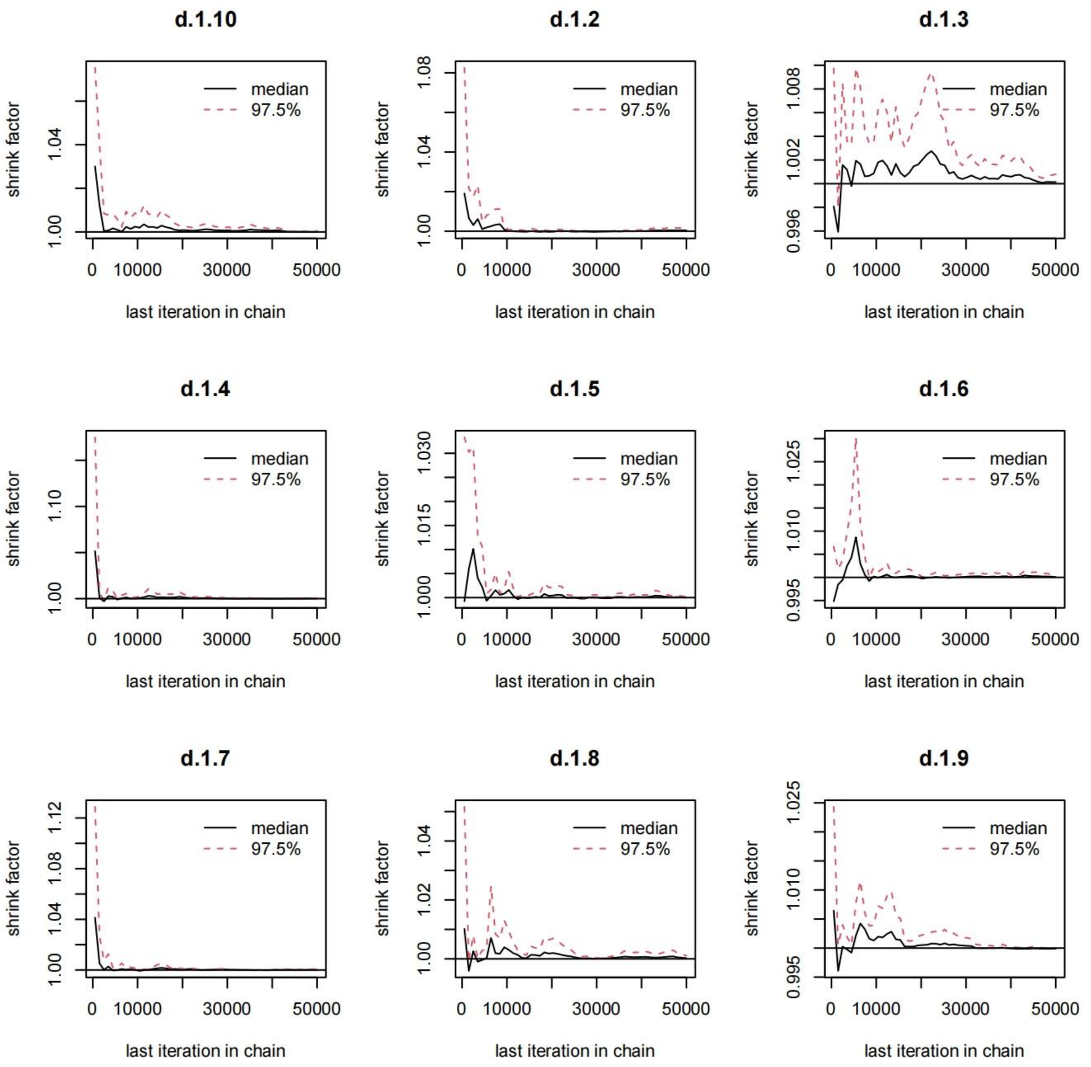

Figure S32.Convergence diagnostics for PFS in HBV-positive advanced hepatocellular carcinoma

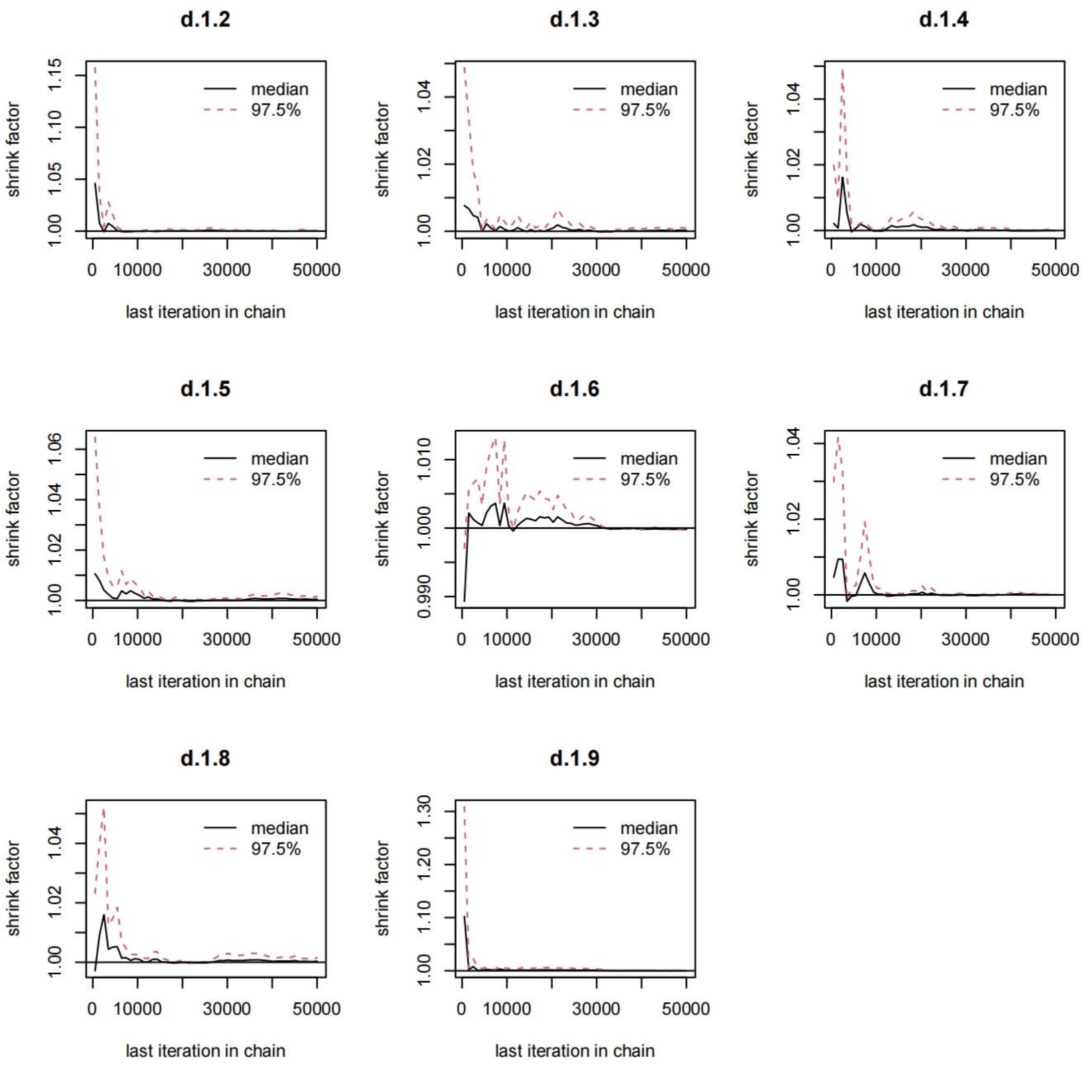

Figure S33.Convergence diagnostics for PFS in HCV-positive advanced hepatocellular carcinoma

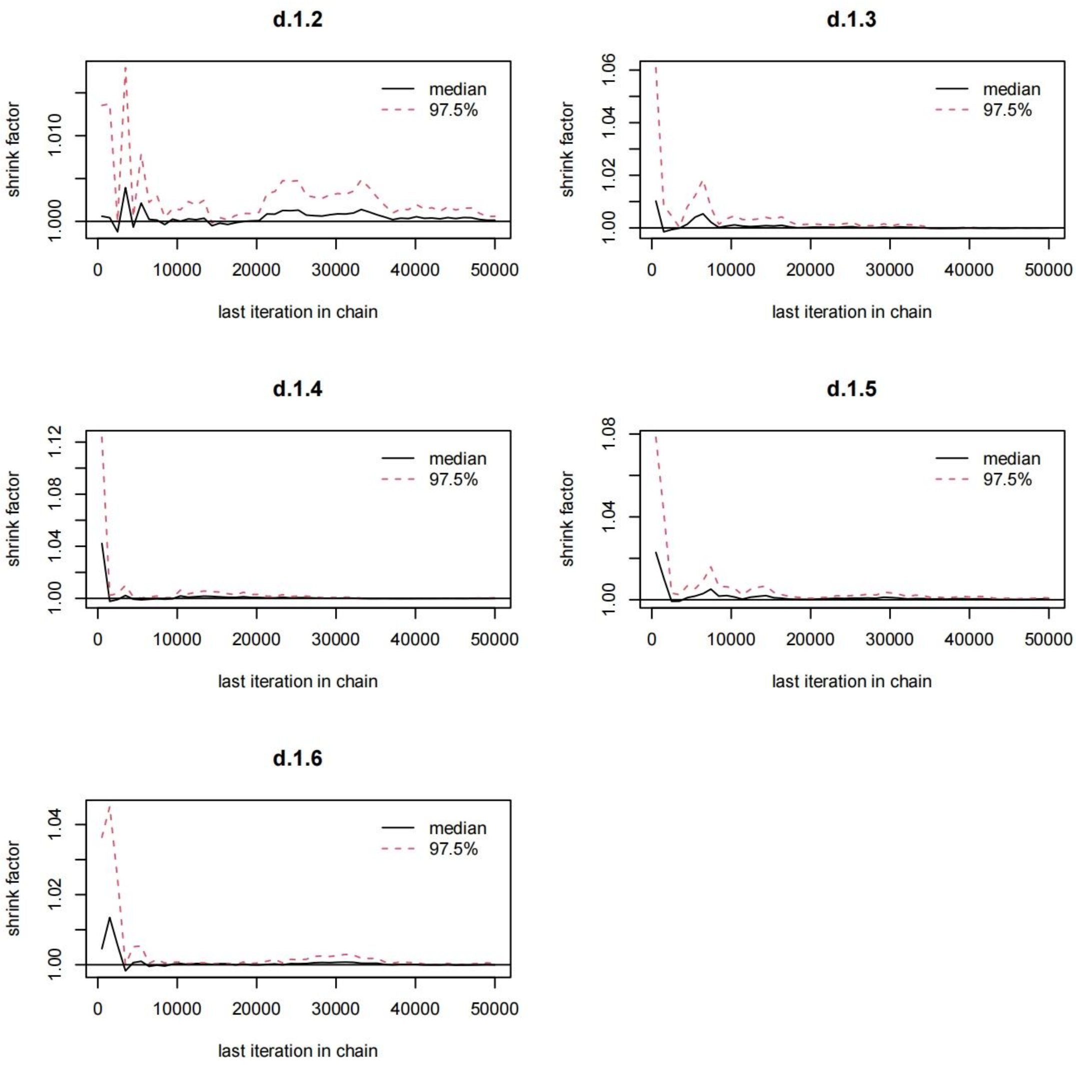

Figure S34.Convergence diagnostics for PFS in NBNC advanced hepatocellular carcinoma
